# Dysbiosis of lower respiratory tract microbiome are associated with inflammation and microbial function variety

**DOI:** 10.1186/s12931-019-1246-0

**Published:** 2019-12-03

**Authors:** Kang-jie Li, Zi-long Chen, Yao Huang, Rui Zhang, Xiao-qian Luan, Ting-ting Lei, Ling Chen

**Affiliations:** 1 School of Public Health and Management, Chongqing Medical University, Chongqing, 400016 China; 2 First Clinical College, Chongqing Medical University, Chongqing, 400016 China; 3 The Center of Experimental Teaching Management, Chongqing Medical University, Chongqing, 401331 China

**Keywords:** Lower respiratory tract, Microbiome, Inflammatory mediators, Function of microbiome, Smoking

## Abstract

**Background:**

Lower respiratory tract (LRT) microbiome has been reported to associate with pulmonary diseases. Unregulated inflammation is an underlying cause of variable lung diseases. The lung microbiome may play an important role in the smoking-induced inflammatory lung diseases. What’s more, the function of microbiome may be more important for understanding how microbes interact with host. Our study aims to explore the effects of smoking on the lower respiratory tract microbiome, the association between variation of lower respiratory tract microbiome and inflammation and whether smoking exposure changes the function of lower respiratory tract microbime.

**Methods:**

Forty male mice were randomly divided into smoking group and non-smoking group, and the smoking group was exposed to cigarette smoke for 2 h per day for 90 days. After experiment, the blood samples were collected to measure the concentration of interleukin-6 (IL-6) and C reactive protein (CRP) by ELISA. Lung tissue samples were used to detect the community and diversity of lower respiratory tract microbiome through 16S rRNA gene quantification and sequencing technology. ANOSIM and STAMP were performed to analyze the differences of the microbial community structure between smoking group and non-smoking group. SPSS 24.0 software was used to analyze the correlations between microbiome and inflammation mediators through scatter plots and Spearman correlation coefficient. Microbial metabolic function was predicted by PICRUSt based on the 16 s rRNA gene quantification and sequencing results. PATRIC database was searched for the potential pathogenic bacteria in lower respiratory tract.

**Results:**

Our results suggested that smoking had markedly effects on the microbiota structure of lower respiratory tract based on Bray-Curtis distance (R^2^ = 0.084, *p* = 0.005) and on unweighted uniFrac distance (R^2^ = 0.131, *p* = 0.002). Smoking mainly affected the abundance of microbiome which belong to *Proteobacteria* phyla and *Firmicutes* phyla. Moreover, our results also found that smoking increased the abundance of *Acinetobacter*, *Bacillus* and *Staphylococcus*, which were defined as pathogenic bacteria. Inflammatory mediators were observed to associate with certain microbiome at every level. Most of microbiome which were associated with inflammation belonged to *Proteobacteria* phyla or *Firmicutes* phyla. Moreover, we found that the decreased microbiome in smoking group, including *Oceanospirillales*, *Desulfuromonadales, Nesterenkonia*, and *Lactobacillaceae,* all were negatively correlated with IL-6 or CRP. Based on the level of inflammation, the abundance of microbiome differs. At genus level, *Lactobacillus*, *Pelagibacterium, Geobacter* and *Zoogloea* were significantly higher in smoking group with lower IL-6 concentration. The abundance of microbiome was not observed any statistical difference in subgroups with different weight. Three dominant genus, defined as pathogen, were found higher in the smoking group. The microbial functional prediction analysis revealed that ABC-type transport systems, transcription factors, amino acide transport and metabolism, arginine and proline metabolism et al. were distinctively decreased in smoking group, while the proportions of replication, recombination and repair, ribosome, DNA repair and recombination proteins were increased in smoking group (q < 0.05).

**Conclusions:**

Members of *Proteobacteria* phyla and *Firmicutes* phyla played an important role in the microbial community composition and keeping a relatively balanced homeostasis. Microbiome dysbiosis might break the balance of immune system to drive lung inflammation. There might exist potential probiotics in lower respiratory tract, such as *Lactobacillaceae*. The altered function of Lower respiratory tract microbiome under smoking exposure may affect the physiological homeostasis of host.

## Background

There exist complex microbial communities in human body and the complementary functions of microbiome are vital to maintain the host physiological homeostasis, such as helping in absorbing nutrition, resisting pathogens, degenerating toxicant and regulating immune system [1, 2]. Recently, the gut microbiome has attracted much attention and even been viewed as a virtual organ, because many studies demonstrate that the gut microbiome could affect the human health in direct or indirect ways [3, 4]. For example, *Helicobacter pylori* contributes to the peptic ulcers and increases the risk of gastric cancer [5]. But due to the conception that the lower respiratory tract (LRT) is sterile, the lung microbiome has received less attention compared with the gut. However, as the rapid development of high-throughput next generation sequencing, culture-independent methods, many studies identify that LRT is colonized by a vast number of microbes which is also distinctive to the microbes of upper respiratory tract [6]. Furthermore, the global change in microbial community structure and changes of abundance and diversity of LRT microbiome are associated with pulmonary disease such as chronic obstructive pulmonary disease (COPD), asthma, cystic fibrosis (CF) and lung cancer [7–9].

Tobacco smoking is one of the prime risk factors for many diseases and linked to 6 million deaths every year [10], but the underlying mechanism remains unclear. We all know that pathogenic bacteria can cause local or systemic inflammatory responses. Equally, smoking can also lead to chronic inflammation [11]. Inflammation played an important role in destroying the invaded pathogenic bacteria and protecting the organisms. But unregulated inflammation is an underlying cause of variable diseases [4, 12]. For example, inflammation of the gut induces carcinogenic mutagenesis and promotes the happen of colorectal cancer [13]. Our previous study shows that higher microbial diversity is observed in the smoking group and some genus are less in smoking group [14]. Accumulating studies show that the lung microbiome are associated with pathology of the respiratory tract. So we hypothesize that the microbiome may play an important role in the smoking-induced inflammatory lung disease. We speculate that microbiome dysbiosis or specific members of LRT microbiome might participate in the processing of host inflammation and promote the development of lung diseases through underlying mechanisms. It is probably due to the poor nutrient status in LRT, the number of microbial communities is relatively lower in healthy lungs [15]. Among, a number of microbiome can not be cultured or classified and the feature and function of these microbiome are also unknown. Moreover, a growing number of studies show that compared with microbial composition, the function of microbiome is more important for understanding how microbe interact with environmental condition and host [16, 17]. The Human Microbiome Project also shows that the function of microbiome remains relatively stable, despite of dramatic variations in the community structure [18]. So it is necessary to explore how the variation of function of LRT microbiome affects the pathological process of lung disease.

In the present study, we established mice models that exposed to cigarette smoke for 90 days to investigate how the microbiome hosted in the LRT evolved under smoking environment and interacted with host in the development of chronic pulmonary disease by using 16S rRNA gene sequencing, Spearman correlation analysis and Phylogenetic Investigation of Communities by Reconstruction of Unobserved States (PICRUSt) functional prediction. We aimed to answer three questions: 1) How smoking shaped the community composition of LRT microbiome? Which microbiome did smoking affect most? 2) Is the variation of LRT microbiome associated with inflammation response? 3) Dose smoking exposure change the function of LRT microbiome?

## Materials and methods

### Study design

Animals used in this experiment are totally 40 male kun-ming mice, 8 weeks old, body weight 20-22 g. All mice were brought from Experimental Animal Center, Chongqing Medical University. Animals were randomly distributed into smoking group and non-smoking group, 20 mice in each group. Mice in smoking were exposed to cigarette (Brand name: ‘Five Cattle’) smoke for consecutive 90 days, with 14 cigarettes/day, 2 h each time. Each cigarette contains 1.0 mg nicotine. Smoke exposure was conducted in the HOPE-MED 8050 Dynamic Poison Control System (From *Hepu* Industry of Tianjin, China) and the non-smoking group only feed with water and fodder [19]. Weight was recorded on Tuesday and Saturday every week, activity and diet were observed everyday. At the end of our experiment, 36 mice survived (with 18 mice in each group). Mice were decapitated in a sterile laboratory to collect blood, then the chest cavity were dissected to obtain the lung tissue. The right and left lung lobe were kept separately. Taking right lung tissue stored in −80 °C until microbiome sequencing conducted. Blood was centrifuged (3500 rpm, 10 min) immediately, serum which obtained and stored in −80 °C were used for ELISA. All animals were treated according to the approved protocols for the BCM Institutional Animal Care and Usage Committee.

### Enzyme linked immunosorbent assay (ELISA) analysis

According to the manufacturer’s instructions, serological analysis was performed to measure serum IL-6 and CRP levels. IL-6 was determined by IL-6 ELISA kits (Cat.#:CK-E20012M,48 T) and CRP was quantified by CRP ELISA kits (Cat.#:CK-E30459M,48 T). All serum samples were obtained from mice and stored at −80 °C. Firstly, standards and samples were added to the microelisa stipulate respectively (50ul standards were added to the standard well, and 10ul testing sample and 40ul sample diluent were added in sample well). Then, added 100ul HPR-conjugate reagent to each well, covered with adhesive strip and incubated for 60 min at 37 °C incubators. Then each well was aspirated and washed by wash solution (400ul), and repeated this process four times for a total of five washes. Chromogen solution A and Chromogen solution B were added to each well and incubated for 15 min at 37 °C in incubators protected from light. Finally, stop solution was added to each well, and optical density (O.D.) was measured at 415 nm by using the standard microplate reader (ELx808). The unit of IL-6 is pg/ml, ng/ml for CRP.

### Analysis of microbial community

Microbial DNA was extracted, amplified, sequenced as previously described [20–22]. Genomic DNA of lung samples was extracted using E.Z.N.A.® Soil DNA Kit (Omega Bio-tek, Norcross, GA, U.S.). For the V4-V5 region of the bacterial 16S ribosomal RNA gene amplifying, the reaction cycle parameters were as follows: 95 °C for 2 min, followed by 25 cycles at 95 °C for 30 s, 55 °C for 30 s, and 72 °C for 30 s and a final extension at 72 °C for 5 min. The used primer sequences were: 515F 5′-barcode-GTGCCAGCMGCCGCGG-3′ and 907R 5′-CCGTCAATTCMTTTRAGTTT-3′ where the barcode was an eight-base sequence unique to each sample. The PCR reactions mixture contained 4 μL of 5 × FastPfu Buffer, 2 μL of 2.5 mM dNTPs, 0.8 μL of each primer (5 μM), 0.4 μL of FastPfu Polymerase, and 10 ng of template DNA. Agarose gels were used for purification of the amplified product with the AxyPrep DNA Gel Extraction Kit (Axygen Biosciences, Union City, CA, U.S.). DNA concentration was measured using QuantiFluor™ -ST (Promega, U.S.).

### Library construction and sequencing

Purified PCR products were determined using Qubit®3.0 (Life Invitrogen). The every 24 resulting amplicons whose barcodes were specific were mixed equally for Illumina Pair-End library construction according to Illumina’s genomic DNA library preparation procedure. Then the paired-end sequenced (2 × 250) on an Illumina HiSeq platform was used for amplicon library based on the manufacturer’s instructions.

### Processing of sequencing data

Raw data was processed using QIIME (version 1.17). Filter Data was obtained after a preliminary analysis of the raw data. And there were following criteria: (1) Filter the base which was truncated at the tail with quality score below 20, and discard the read with quality below 50 bp. (2) Permitting barcode matching is 0, maximum primer mismatch is 2 and ambiguous reading characters all were deleted. (3) Only sequences that overlap longer than 10 bp could be assembled with their overlap sequence, reads that could not be assembled were removed.

### Statistical analysis

According to the Standard Operating Procedure, inflammatory mediators were analyzed using SAS 9.1.which indicated a minimum sequence length of 250 bp was used to MiSeq sequence [23, 24], sequenced data were processed and analyzed using Mothur v.1.21.1 [25]. *P* < 0.05 was considered a statistical significance. Operational taxonomic units (OTUs) were defined at a cutoff of 97% with the use of UPARSE 7.1 and UCHIME was used to select chimeric sequences. After filtering of the chimeric sequences, bacterial community analyses based on 16S rRNA genes were performed using RDP Classifier (http://rdp.cme.msu.edu/) against the silva (SSU129)16S rRNA database with confidence threshold of 70% [26].

Effect of smoking on the microbial community structure was reflected by ANOSIM which contained unweighted UniFrac distance and Bray-Curtis distance. *P-value< 0.05 was noted to a significant difference. STAMP was used to analyze the significant difference microbial community and the main reason. *P*-value <0.05 was considered as statistical significance. Spss24.0 (IBM Corp, Armonk, NY, USA) was used to analyze the correlation between inflammatory mediators and microbial community. Data was expressed as percent or mean ± standard deviation (SD). The correlations between microbiome and IL-6 or CRP were analyzed by making scatter plots and Spearman bivariate correlation. *P*-value <0.05 was considered as an indication of significant difference. The *t*-test was performed to analyze the difference of microbiome in two independent groups. The microbial metabolic function was predicted by PICRUSt with three KEEG levels which based on 16S rRNA gene sequence [27]. PATRIC database was searched for potential pathogenic bacteria in lower respiratory tract.

## Result

### Microbial community profiles in the lower respiratory

A total of 1879 Operational Taxonomic Units (OTUs) were identified with the criterion of 97% sequence similarity. The OTU distribution of the LRT microbiome revealed 157 OUTs were observed in more than 50% samples and 37 OUTs in all samples. These OUTs were considered core microbiome (Additional file 1: Figure S1). Microbiome can be divided into phyla, class, order, family, genus, and species according to the level, and our analysis are also based on the levels. We further investigated the top 30 genus which mainly belong to the *Proteobacteria*, *Firmicutes* and *Actinobacteria*. *Halomonas* (20.99%), *Sphingomonas* (6.21%), *Lactobacillus* (5.52%), *Pelagibacterium* (4.51%) were the dominant genus in both smoking and non-smoking group (Additional file 1: Figure S2).

### Effect of smoking on the microbial community structure in lower respiratory tract

To reveal the impact of smoking on the microbiota structure, we computed the distance within groups or among groups. The boxplot showed a higher dissimilarity within smoking samples than within non-smoking samples whether based on Bray-Curtis distance or based on (unweighted / weight) uniFrac distance (Additional file 1: Figure S3. A, B, C). For qualitative result, we further used Anosim analysis to discuss the variation of microbial community structure (Additional file 2: Table S1). The result revealed that the microbial community structure of the smoking group was significantly different from that of non-smoking group based on Bray-Curtis distance (*R*^2^ = 0.084, *p* = 0.005) and on unweighted uniFrac distance (*R*^2^ = 0.131, *p* = 0.002). However, the differences were not significant based on weighted uniFrac distance (*R*^2^ = 0.019, *p* = 0.202). These results suggested that smoking had markedly effects on the microbiota structure of lower respiratory tract and the smoking group tended to show greater individual differences.

### Effect of smoking on the microbial composition in lower respiratory tract

Besides the overall microbial community structure, we also explored the change of microbial composition in lower respiratory tract under smoking exposure. At phylum level, after filtering the relative abundance lower than 0.2% in any groups, 7 phyla were identified. *Proteobacteria, Firmicutes, Actinobacteria and Bacteroidetes* were the dominant microbial phyla (the relative abundance >1%). There were no significant differences between two groups (*P* > 0.05) (Additional file 2: Table S2). At class level, after filtering the relative abundance lower than 0.2% in any groups, 15 classes were identified. *Gammaproteobacteria, Alphaproteobacteria, Bacilli, Betaproteobacteria, Clostridia and Actinobacteria* (the relative abundance >5%) were the predominant classes in two groups, but smoking did not distinctly affect these dominant classes (*P* > 0.05). However, the relative abundance of *Deltaproteobacteria* (1.13% vs. 1.43%) and *Chloroplast* (0.55% vs. 1.53%) significantly decreased in smoking group (*P* < 0.05) (Additional file 2: Table S3). The effects of smoking exposure on lower respiratory tract microbiome require more data from other levels.

In order to investigate what kinds of bacteria in lower respiratory tract was affected by smoking. We further analyzed the composition from order to species level. The relative abundance lower than 0.5% in any groups were filtered, a total of 22 orders and 35 families were identified. *Oceanospirillales, Lactobacillales, Rhizobiales, Clostridiales, Sphingomonadales, Pseudomonadales, Burkholderiales, Bacillales* were dominant orders, and *Halomonadaceae, Sphingomonadaceae, Moraxellaceae* and *Lactobacillaceae* were four dominant families (the relative abundance >5%) (Additional file 2: Table S4, S5). The result showed that smoking decreased *Oceanospirillales, Enterobacteriales, Desulfuromonadales* and *Chloroplast_norank* ratio at order lever (*P* < 0.05). Furthermore, seven families including *Lactobacillaceae, Enterobacteriaceae*, *Phyllobacteriaceae, Geobacteraceae, Chloroplast_norank, Oxalobacteraceae* and *Burkholderiaceae* in smoking group were markedly different from Non-smoking group (*P* < 0.05). Among the seven families, the abundance of *Oxalobacteraceae* in smoking group was significantly increased, but the other six families in smoking group were significantly decreased.

On the genus level, after filtering the relative abundance lower than 0.5% in any groups, 34 genus were detected. The most abundant genus were *Halomonas, Sphingomonas* and *Lactobacillus* (>5% abundance). We further found that many genus were significantly decreased in smoking group including *Lactobacillus, Kluyvera, Nesterenkonia* and *Mesorhizobium* et al., but there were only two genera increased including *Trichococcus* and *Escherichia-Shigella* (*P* < 0.05) (Additional file 2: Table S6). Similar to the above comparison in the genus level, a total of 37 species were detected. Eight species decreased in smoking group such as *Streptococcus gallolyticus subsp.macedonicus, kluyvera ascorbata* and *Mesorhizobium_Unclassified* while only 3 species increased in smoking group including *Streptococcus_uncultured bacterium, Trichococcus_uncultured bacterium* and *Escherichia-Shigella_Unclassified* (*P* < 0.05) (Additional file 2: Table S7).

We also concluded the classification of microbe with significant changes (Table 1). The results showed that smoking mainly affected microbe belonging to the *Enterobacteriales, Desulfuromonadales* and *Phyllobacteriaceae,* which all were members of *Proteobacteria* phyla. Smoking also influenced the abundance of microbe classified as the *Lactobacillaceae* and *Streptococcus,* which were members of *Firmicutes* phyla*.*

**Table 1:** Taxonomy of microbe with significant changes in two groups

Phylum	Class	Order	Family	Genus	Species
Proteobacteria	Gammaproteobacteria	**Oceanospirillales**			
		**Enterobacteriales**	**Enterobacteriaceae**	**Kluyvera**	**Kluyvera ascorbata**
				**Escherichia-Shigella**	**Escherichia-Shigella_Unclassified**
				**Enterobacter**	**Enterobacter_Unclassified**
				**Raoultella**	**Raoultella_Unclassified**
	**Deltaproteobacteria**	**Desulfuromonadales**	**Geobacteraceae**	**Geobacter**	
	Betaproteobacteria	Burkholderiales	**Oxalobacteraceae**		
			**Burkholderiaceae**		
	Alphaproteobacteria	Rhizobiales	**Phyllobacteriaceae**	**Mesorhizobium**	**Mesorhizobium_Unclassified**
				**Phyllobacteriaceae_uncultured**	**Phyllobacteriaceae_uncultured bacterium**
		Caulobacterales	Caulobacteraceae	**Caulobacteraceae_Unclassified**	**Caulobacteraceae_Unclassified**
Firmicutes	Bacilli	Lactobacillales	Streptococcaceae	Streptococcus	***Streptococcus gallolyticus subsp. macedonicus***
					**Streptococcus_uncultured bacterium**
			Carnobacteriaceae	**Trichococcus**	**Trichococcus_uncultured bacterium**
			**Lactobacillaceae**	**Lactobacillus**	
		Bacillales	Paenibacillaceae	**Brevibacillus**	
	Clostridia	Clostridiales	Clostridiaceae 1	**Clostridium sensu stricto 6**	
Cyanobacteria	**Chloroplast**	**Chloroplast_norank**	**Chloroplast_norank**	**Chloroplast_norank**	**Chloroplast_Unclassified**
Actinobacteria	Actinobacteria	Micrococcales	Micrococcaceae	**Nesterenkonia**	

The bold microbiome are statistically different between smoking group and non-smoking group

Finally, we examined the potential pathogenic bacteria in mice. PATRIC database records pathogen bacteria of humans. Since microbiome communities in LRT are similar between humans and mice, we also examined potential pathogenic genus by PATRIC database. Three genus were defined as pathogenic bacteria among the dominant genus, including *Acinetobacter*, *Bacillus* and *Staphylococcus*. Although without statistical difference, these 3 genus stated a higher abundance in smoking group than non-smoking group. It indicated smoking increased some pathogen bacteria.

### The association between variation of LRT microbiome and inflammation

To explore the relationship among smoking, variation of LRT microbiome and inflammation in mice, we chose two main inflammatory mediators (IL-6 and CRP). Smoking increased inflammation although without statistical difference.

We further discussed the association between inflammatory mediators and variation of LRT microbiome from phyla to species level. Correlation coefficients were shown in Additional file 2: Table S8-S13. At phylum level, *Acidobacteria* and *Planctomycetes* in smoking group showed strong correlation with CRP. No obvious correlation was observed between IL-6 and other phylum in smoking group. As for non-smoking group, there existed no correlation between inflammatory mediators and any phylum. At Class level, only *Deltaproteobacteria* in smoking group was strongly correlated with IL-6 and the correlation was not obvious for CRP. Still, there was no any correlation between inflammatory mediators and Class members in non-smoking group. Three Order members in smoking group, including *Oceanospirillales, Desulfuromonadales* and *Rhodocyclales*, were observed to correlate with IL-6. Four Family members in smoking group, including *Halomonadaceae, Lactobacillaceae, Geobacteraceae* and *Rhodocyclaceae*, showed strong correlations with IL-6 and the *Ruminococcaceae* Family in smoking group was correlated with CRP. No correlations were observed in non-smoking group at Order or Family level.

At genus level, *Nesterenkonia* and *Geobacter* showed strong correlation with IL-6 and *Clostridium* sensu stricto *1* was correlated with CRP in smoking group, while *Lactobacillus* showed the strongest correlations with both IL-6 and CRP. None of the genus showed any correlation with IL--6 or CRP in non-smoking group. At Species level, four microbiome, including *Halomonas_Unclassified, Lactobacillus_uncultured bacterium, Nesterenkonia sp. NP1,* and *Geobacter_Unclassified,* were observed to correlate with IL-6 in smoking group*. Nesterenkonia sp. NP1* in non-smoking group also showed correlation with IL-6, however the differences of *Nesterenkonia sp. NP1* between the two groups were not statistically significant.

**Fig. 1:**
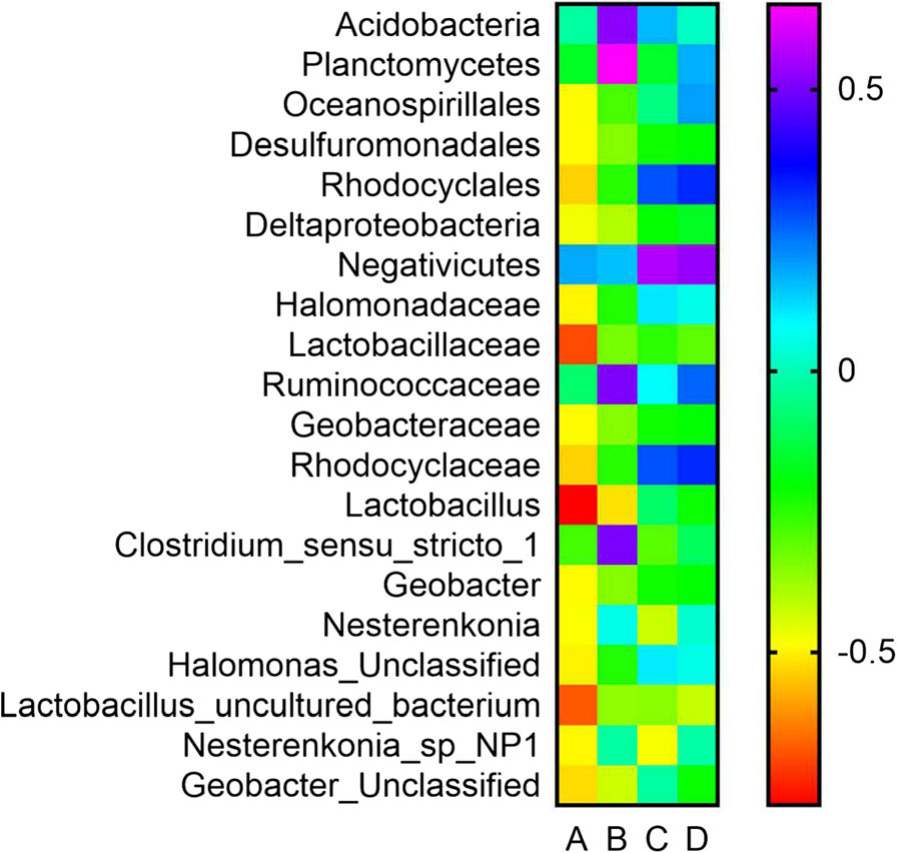
Heat map of correlation coefficient value. Column **A** represents the correlation between IL-6 and microbiome in smoking group. Column **B** represents the correlation between CRP and microbiome in smoking group. Column **C** represents the correlation between IL-6 and microbiome in non-smoking group. Column **D** represents the correlation between CRP and microbiome in non-smoking group. Twenty microbiome which significantly associate with any inflammatory mediator in either smoking group or non-smoking group are stated

We also concluded the taxonomy of these microbiome that correlated with inflammatory mediators in the Table 2. We found that these microbiome were relative with each other, for example, the *Geobacter_Unclassified* Species was one of *Geobacter* Genus, the *Geobacter* was a member of *Geobacteraceae* Family, the *Geobacteraceae* was classified into *Desulfuromonadales* Order and the *Desulfuromonadales* belonged to *Deltaproteobacteria* Class, the five microbiome were all strongly correlated with inflammation factors and they finally were classified into *Proteobacteria* Phyla. From phyla to species level, a total of 19 microbiome in the smoking group was associated with inflammatory mediators. Among them, five microbiome were members of *Firmicutes* Phyla and ten microbiome were classified into *Proteobacteria* Phyla. These results demonstrated that *Firmicutes* and *Proteobacteria* Phyla played an important role in the micro-ecology of lower respiratory tract of mice and in the development of lung inflammatory diseases. Figure 1 depicted the significant correlation coefficients between inflammatory mediators and microbiome by heat map.

**Table 2:** Taxonomy of microbiome that correlated with inflammatory mediators

Species	Genus	Family	Order	Class	Phylum
**Halomonas_Unclassified**	Halomonas	Halomonadaceae	Oceanospirillales	Gammaproteobacteria	Proteobacteria
**Lactobacillus_uncultured bacterium**	Lactobacillus	Lactobacillaceae	Lactobacillales	Bacilli	Firmicutes
**Nesterenkonia sp. NP1**	Nesterenkonia				Actinobacteria
**Geobacter_Unclassified**	Geobacter	Geobacteraceae	Desulfuromonadales	Deltaproteobacteria	Proteobacteria
	**Lactobacillus**	Lactobacillaceae	Lactobacillales	Bacilli	Firmicutes
	**Clostridium sensu stricto 1**	Clostridiaceae	Clostridiales	Clostridia	Firmicutes
	**Nesterenkonia**				Actinobacteria
	**Geobacter**	Geobacteraceae	Desulfuromonadales	Deltaproteobacteria	Proteobacteria
		**Halomonadaceae**	Oceanospirillales	Gammaproteobacteria	Proteobacteria
		**Lactobacillaceae**	Lactobacillales	Bacilli	Firmicutes
		**Ruminococcaceae**	Clostridiales	Clostridia	Firmicutes
		**Geobacteraceae**	Desulfuromonadales	Deltaproteobacteria	Proteobacteria
		**Rhodocyclaceae**	Rhodocyclales	Betaproteobacteria	Proteobacteria
			**Rhodocyclales**	Betaproteobacteria	Proteobacteria
			**Desulfuromonadales**	Deltaproteobacteria	Proteobacteria
			**Oceanospirillales**	Gammaproteobacteria	Proteobacteria
				**Deltaproteobacteria**	Proteobacteria
					**Acidobacteria**
					**Planctomycetes**

The bold microbiome are associated with inflammatory mediators in the smoking group of this study

Furthermore, our analysis also found that all the microbiome that associated with inflammatory mediators in smoking group, except *Acidobacteria, Planctomycetes, Ruminococcaceae,* and *Clostridium* sensu stricto *1,* were negatively correlated with IL-6 or CRP. Interestingly, their relative abundance was lower than the non-smoking group with statistical difference or not, further demonstrating that the dysbiosis of lower respiratory tract microbiome contributed to inflammation. There were seven microbiome correlated with inflammatory mediators and their relative abundance also existed statistical difference between two groups. Additional file 1: Figure S4-S11 indicated that the concentration of IL-6 or CRP increased with the decreasing of these microbiome. These results suggested that changes of microbiome in relative abundance and diversity in lower respiratory tract of mice might have strong relationship with the level of inflammation. In addition, these processing might played an important role in the development of respiratory diseases.

### Microbiome differs based on inflammation levels

Study showed that prebiotics shaped the level of inflammation and the composition of lower respiratory tract microbiome, indicating a potential connection between inflammation levels and composition of the microbiome. To figure out whether the relative abundance of microbiome differs according to the level of inflammation, we compared the difference of dominant microbiome at genus level in smoking group. The total 18 samples were divided into two subgroups based on the median concentration of IL-6 (8.31 pg/ml). The results depicted that *Lactobacillus* (*P* = 0.002), *Pelagibacterium* (*P* = 0.022), *Geobacter* (*P* = 0.043) and *Zoogloea* (*P* = 0.033) were significantly higher in the subgroup with lower IL-6 concentration while no statistical differences were observed in other genus. Besides, except *Lactobacillus* belongs to *Firmicutes* phylum, the other three genus are members of *Proteobacteria* phylum. In the non-smoking group, only *Raoultella*, one of *Proteobacteria* phylum, showed higher relative abundance in the subgroup with higher IL-6 concentration (*P* = 0.001).

### Weight and microbiome composition

Physical conditions of mice may affect the diversity or composition of lower respiratory tract microbiome, such as weight. So we conducted an analysis to explore the possible relation between weight and abundance of dominant genus. The 18 samples in non-smoking group were divided into two subgroups based on the average weight. However, there were no statistical differences in any genus, which might due to the small sample and unobvious variance in weight.

### Microbiota function prediction in lower respiratory tract

**Fig. 2:**
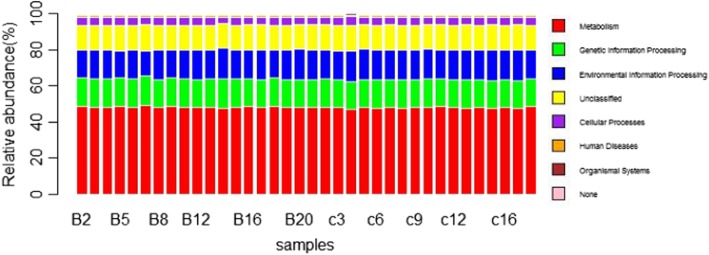
Relative abundance of function at KEGG Level1. Samples B2 to B20 belong to the smoking group and samples c3 to c16 belong to the non-smoking group

Previous studies show that the function of microbiota is more important for understanding how microbe inhabiting in human body influence the health of human except microbial composition. Hence, we predicted the functional profiles in LRT microbiota by using PICRUSt as a predictive exploratory tool. At KEGG1 (Kyoto Encyclopedia of Genes and Genomes) level, the metabolism was the most abundant in both groups (smoking VS non-smoking: 48.3% VS 47.9% respectively) (Fig. 2).

**Fig. 3:**
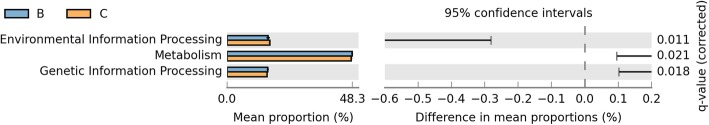
Difference of metabolism, genetic information processing and environmental information processing in two groups (level 1). Group B colored with blue represents the smoking group and group C colored with yellow represents the non-smoking group

**Fig. 4:**
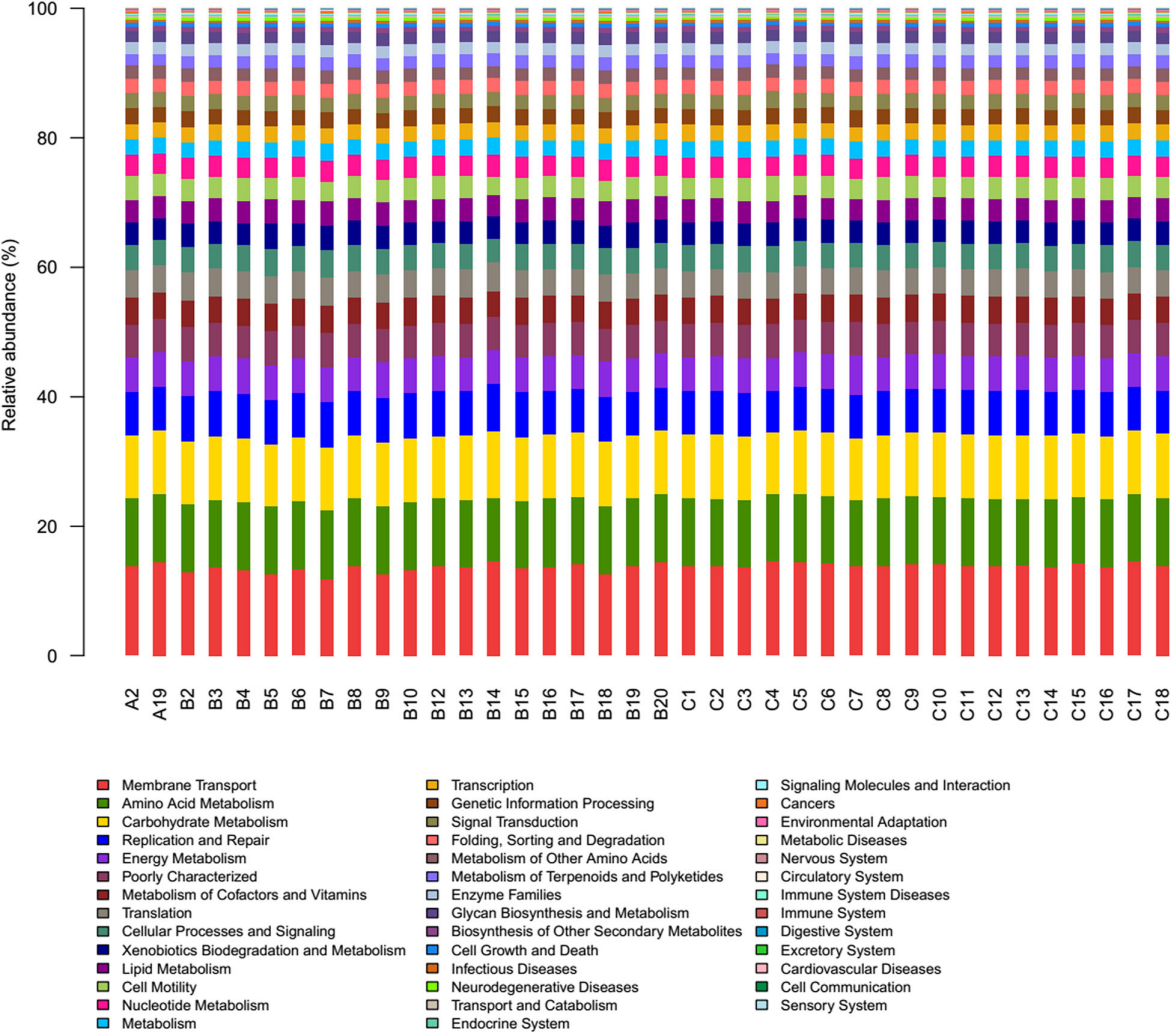
41 level 2 KEGG Orthology groups in smoking and non-smoking groups. Samples A2 to B20 belong to the smoking group and samples C1 to C18 belong to the non-smoking group

**Fig. 5:**
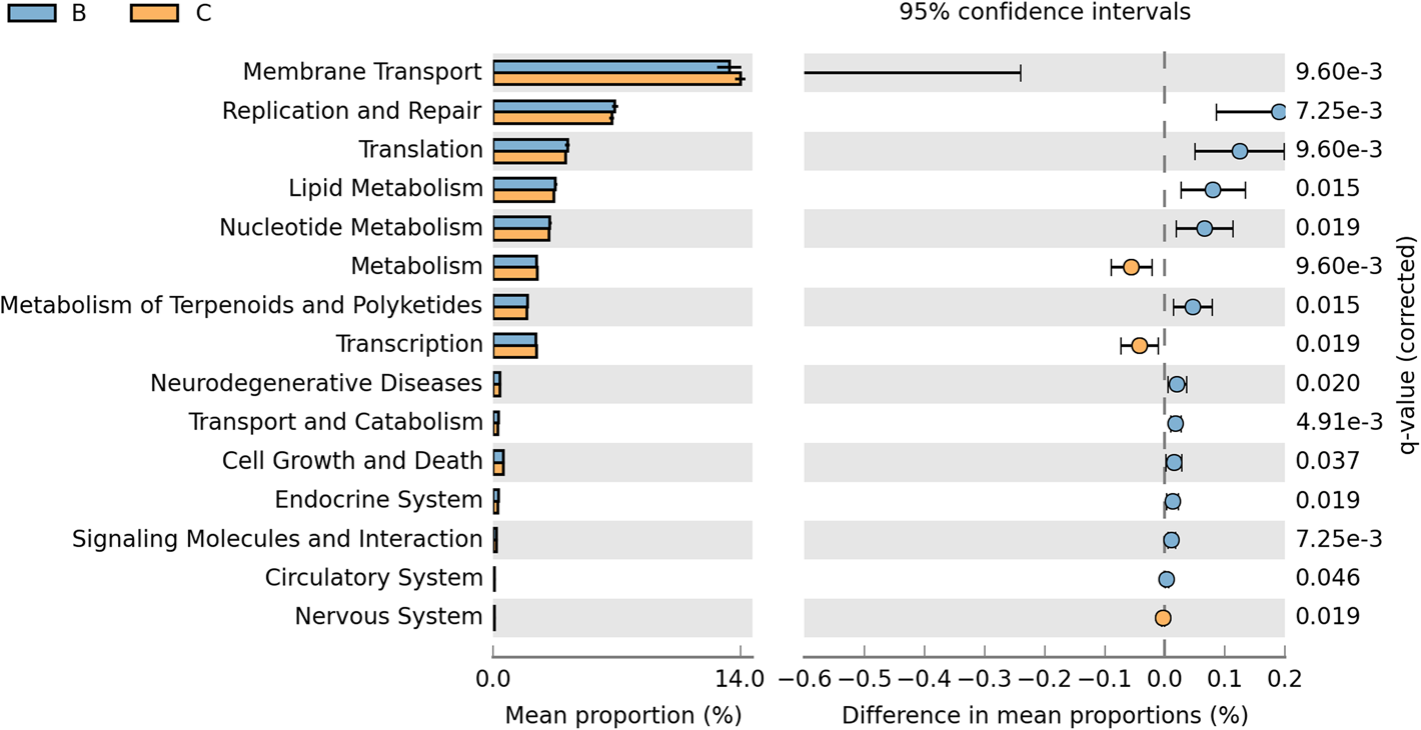
Differences among the 41 KEGG2 pathway between two groups. Group B colored with blue represents the smoking group and group C colored with yellow represents the non-smoking group

In addition, we found that there exited difference between two groups in pathway about metabolism, genetic information processing and environmental information processing (q < 0.05, Fig. 3). Furthermore, a total of 41 level-2 KEGG Orthology groups were showed in the Fig. 4, and the major pathways were membrane Transport, Amino Acid Metabolism and Carbohydrate Metabolism. Among the 41 KEGG2 pathway,

we found that the proportions of 4 pathway (Membrane Transport, Metabolism, Transcription and Nervous System) were decreased in smoking group and 11 pathways were significantly enriched in smoking group including replication and repair, translation, lipid metabolism, nucleotide metabolism, metabolism of terpenoids and polyketides and et al. (q < 0.05, Fig. 5).

**Fig. 6:**
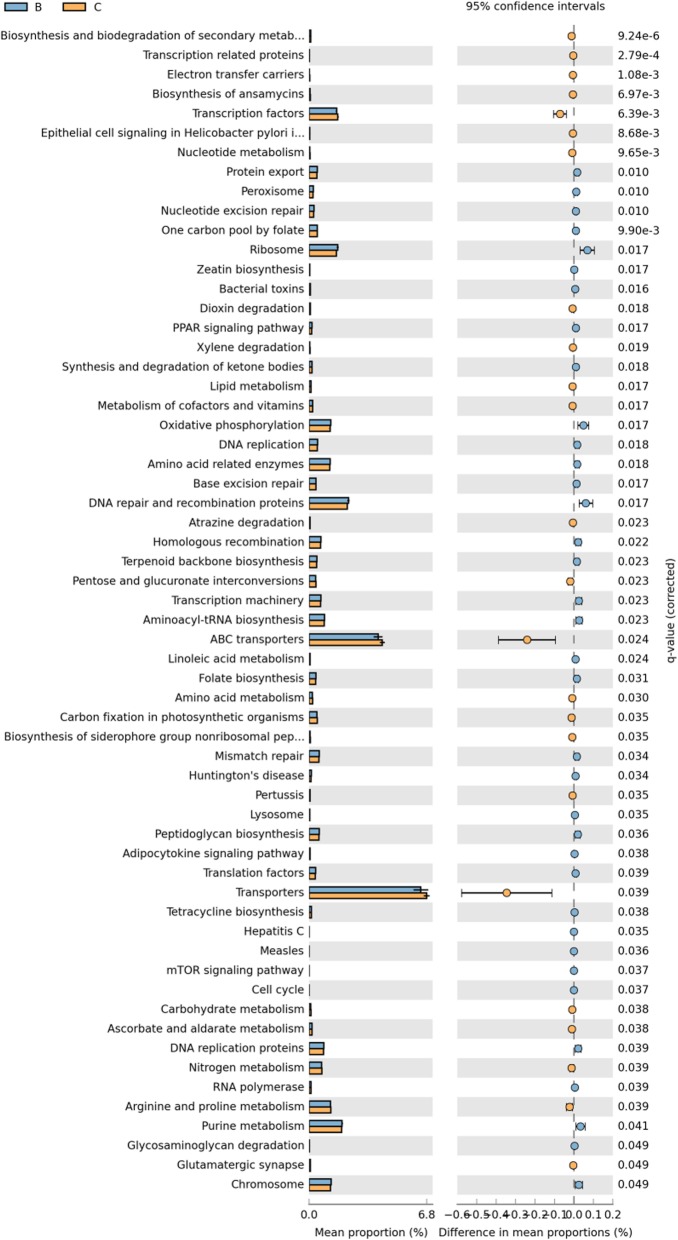
The differences of metabolic pathways at KEGG level 3 between two groups. Group B colored with blue represents the smoking group and group C colored with yellow represents the non-smoking group

We further analyzed the differences of metabolic pathways at KEGG level-3 between two groups. There were 60 pathways exited differences between two groups. 24 KEGG3 pathways, including transporters, ABC transporters, transcription factors, arginine and proline metabolism and so on were decreased in smoking group, while the abundance of 36 pathways were significantly increased in smoking group such as ribosome, DNA repair and recombination proteins, oxidative phosphorylation, purine metabolism, pyrimidine metabolism and et al.(q < 0.05, Fig. 6).

**Fig. 7:**
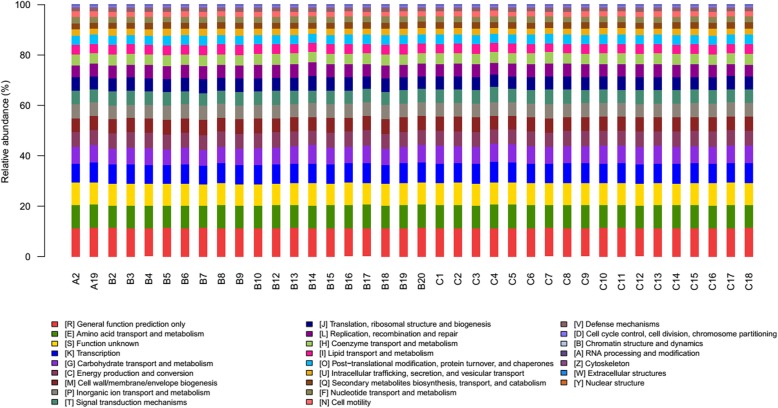
The category of General function prediction at COG level2. Samples A2 to B20 belong to the smoking group and samples C1 to C18 belong to the non-smoking group

**Fig. 8:**
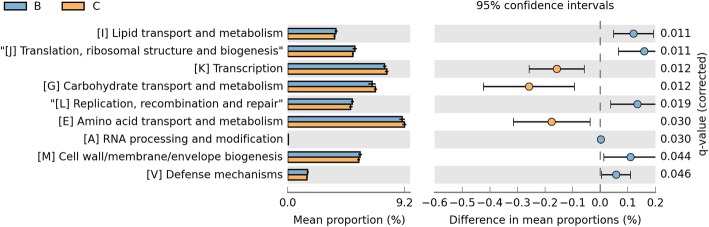
The difference of specific functions between two groups at COG level2. Group B colored with blue represents the smoking group and group C colored with yellow represents the non-smoking group

**Fig. 9:**
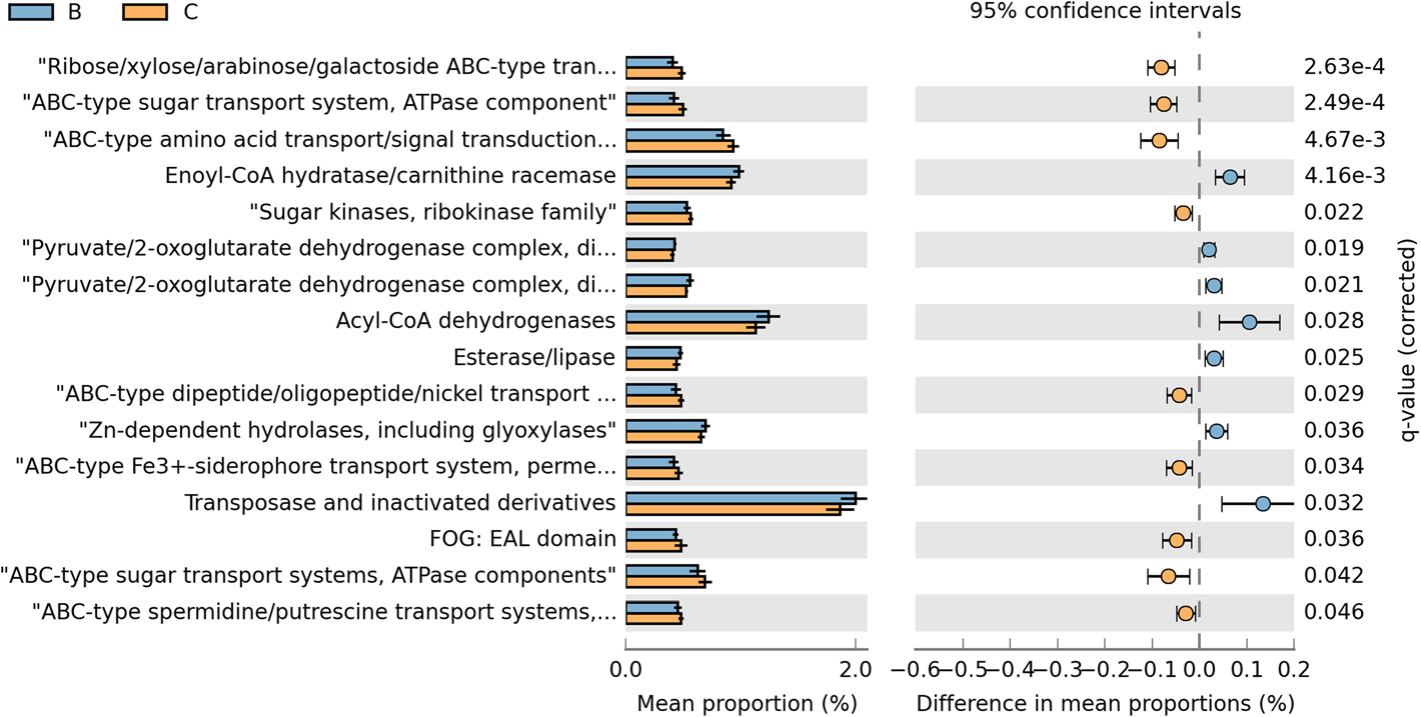
Difference of the specific function of proteins in two groups. Group B colored with blue represents the smoking group and group C colored with yellow represents the non-smoking group

Other for KEGG pathway, we also explored the effect of smoking on LRT microbita in COG (Clusters of Orthologous Groups of proteins) function classification in order to discover the metabolic pathways and key protein that have changed in smoking environment. The result showed that at COG level-2, the category of General function prediction only, Amino acid transport and metabolism, Transcription, Carbohydrate transport and metabolism were the dominant function (Fig. 7). In addition, we found that the function related to lipid transport and metabolism, translation, ribosomal structure and biogenesis, Replication, recombination and repair, RNA processing and modification, Cell wall/membrane/envelope biogenesis and defense mechanisms were distinctively increased in smoking group, meanwhile, the proportion of transcription, carbohydrate transport and metabolism and amino acid transport and metabolism were decreased (q < 0.05, Fig. 8), which was similar to the results at KEGG levels. We further analyzed the specific function of proteins by COG function classification and 136 kinds of proteins were identified (the relative abundance >0.1%, Fig. 9). Compared with non-smoking group, 7 functional proteins, including Enoyl-CoA hydratase/carnithine racemase, Pyruvate/2-oxoglutarate dehydrogenase complex, dihydrolipoamide dehydrogenase (E3/E2) component, and related enzymes, Acyl-CoA dehydrogenases, Esterase/lipase and so on were increased in smoking group, while 9 kinds of proteins were decreased especially about Ribose/xylose/arabinose/galactoside ABC-type transport systems (permease components), ABC-type sugar transport system (ATPase components), ABC-type amino acid transport/signal transduction systems (periplasmic component/domain).

## Discussion

As the viewpoint that the lower respiratory tract (LRT) is sterile was challenged, several studies show that the lung is a mucosal tissue harbored by a variety of bacterial community although in normal physiological conditions [28, 29]. In addition, with the rapid development of high-throughput next generation sequencing, many studies suggest that global change in microbial community structure and changes of abundance and diversity of microbiome may play essential roles in the development of human disease through various biological pathways [30, 31]. For example, there are studies showing that transient incursions from Streptococcus are good predictors of asthma [25], while some fermentative anaerobes are important to cystic fibrosis (CF) exacerbations [32]. Tobacco smoking is linked to 6 million deaths annually, 30% of which are due to cancer [10]. Additionally, negative health effects of secondhand smoke are also documented, including lung cancer, cardiovascular disease, asthma, and other respiratory diseases [33, 34]. Hence, in order to find out the potential key bacteria and new pathogenesis of pulmonary disease, we analyzed the impact of smoke exposure on the microbial community composition and function in lower respiratory tract of mice and explored the relationship between LRT microbiome and inflammation.

Different from the general samples including sputum samples, tracheal aspirates and bronchoalveolar lavage samples, we directly removed the lungs under sterile conditions to avoid the contamination of mouth and upper respiratory tract. And by high-throughput sequencing of 16S rRNA genes, we observed that *Proteobacteria, Firmicutes and Actinobacteria* were the dominant phyla, which accorded with previous studies [35], but in our study the predominant genus were *Halomonas, Sphingomonas, Lactobacillus and Pelagibacterium* in LRT of mice while Nisha Singh et al. [35] reports that *Lactobacillus, Veillonella, Achromobacter* and *Streptococcus* are the most abundant genus in the mouse of 8 weeks and Kenneth KB et al. [36] shows *Staphylococcus, Massilia, Corynebacterium* and *Pseudomonas* are the major genus. This suggested the prime microbiome were the same in LRT of mice at phyla level but different at genus level which may result from different mouse species, age, gender, weight or different living environment.

To the best our knowledge, there were limited studies having utilized the animal models that exposed to cigarette smoke for 90 days to implicate the contribution of LRT microbiome in lung pathologies. In the present study, the result showed that the inter-individual LRT microbiome community structure was more different in smoking group and more similar in Non-smoking group. A possible explanation is that under smoking condition, the need of catabolism and anabolism increased, resulting the change of microbial composition or abundance. The lung microbiome is shown to be associated with both the physiology and pathology of the respiratory tract. For example, there exist associations between the composition and diversity of the LRT microbiome and the process of alveolarization, specifically; *Lactobacillus* might play an important role in lung growth and alveolarization [35]. In addition, Shu MT et al. shows that early asymptomatic colonization with *Streptococcus* is a strong asthma predictor [25]. Studies also demonstrate that during COPD exacerbations, some genera including *Satreptococcus*, *Pseudomonas* and *Haemophilus* changed [37] . Clinical studies suggest that infections with tuberculosis or pneumonia increase the risk of lung cancer [38]. Moreover, Liu HX studied the samples from lung tissues and bronchoscopy and they found that *Streptococcus* was more abundant in cancer cases whereas *Staphylococcus* was more abundant in the controls [39]. In our study, although the dominant microbial phyla exited no significant difference between two groups, smoking still affected some microbe mainly belonging to *Proteobacteria and Firmicutes*. To be specifically, under smoking environment, the abundance of *Enterobacteriales, Desulfuromonadales*, *Phyllobacteriaceae, Lactobacillaceae* and *Chloroplast* were significantly decreased whereas *Trichococcus, Oxalobacteraceae, Escherichia-Shigella* and *Streptococcus_uncultured bacterium* were increased when compared with Non-smoking group. These results further showed that there exited microbiome dysbiosis in the LRT of mouse when exposed to smoking. But the underlying mechanisms of how the dysbiosis happened and the possible mechanisms linking microbiome with the development of lung disease were not entirely understood.

Inflammation plays an important role in destroying the invaded microbes and protecting the organisms [40], but unregulated inflammation is an underlying cause of many chronic diseases such as asthma and chronic obstructive pulmonary disease. Moreover, smoking also can cause chronic inflammation. So we hypothesize that there exist correlation between inflammation and microbiome in lower respiratory tract. Previous studies indicate that alteration of lung microbiome contributes to pulmonary inflammation and participates in the process of airway inflammatory diseases. Barfod et al. [41] provoked lung inflammation in mice by exposure to carbon nano-tube particles, their result showed that induced lung inflammation changed the lung microbiome. Our findings demonstrated that smoking affected the relative abundance of certain dominant microbiome and inflammation mediators were associated with parts of dominant microbiome in lower respiratory tract of smoking group, which suggesting that inflammation might impair the structure of growth environment of microbiome and the microbiome dysbiosis might in turn promoted the inflammation. However, it was still unclear whether certain members of lung microbiome lead to the progress of pulmonary diseases or protected the organisms [42]. Many studies report that some members of *Gammaproteobacteria* in lungs of human and mice are observed to increase during chronic pulmonary diseases [43–45], which may be due to benefit from mucosal inflammation by utilizing inflammatory byproducts to survive in anaerobic and low oxygen conditions [46–48]. Furthermore, *Gammaproteobacteria* also can produce molecular components that promote inflammation. It is also reported that *P.aeruginosa* infection is associated with airway inflammation and poorer prognosis [49]. However, our study showed that the decreased microbiome in smoking group, *Oceanospirillales* belonging to *Gammaproteobacteria* and *Desulfuromonadales* belonging to *Deltaproteobacteria* and *Nesterenkonia* belong to *Actinobacteria* and *Lactobacillaceae,* all were negatively correlated with IL-6 or CRP, which might indicate that decreasing of these microbiome in LRT were positive with inflammation. In other words, these microbiome might play a key role in maintaining the normal function of lung such as structural barriers and the immune barriers. Noteworthy, the *Lactobacillaceae* is proved to protect against pathogens with the capable of anti-inflammatory activities in human gut and also reported for treating autism, alcohol liver disease and alzheimer [50, 51]. So our result indicated that *Lactobacillaceae* might be a potential agent for treating microbiome dysbiosis or chronic inflammation in lung. In order to understand the mechanism of how microbiome of the lower respiratory tract interacted with host lung, many animal models have been developed. Wang XK established microbiome dysbiosis mouse model, they found that microbiome dysbiosis may activate innate lymphoid cells (ILC) and Th2 cell which leads to the imbalance of Th1/Th2, and in turn promotes the development of allergic airway diseases [52]. Furthermore, Gollwitzer et al. [53] established House Dust Mite induced asthma mouse model and demonstrated that microbial signals in the lung of neonatal mice enhanced immune tolerance to House Dust Mite (HDM) allergens via PD-1/PD-L1 signaling in regulatory T cells and dendritic cells (DC). However, Jing Wang evaluated the impacts of treatment with inhaled interferon-γ (IFN-γ) in idiopathic pulmonary fibrosis (IPF) on LRT microbiome and host immune phenotype [54]. Their results demonstrated that IFN-γ didn’t change the LRT microbiome of IPF and the lung microbiome was independently correlated with host immunity. The link between chronic inflammation and cancer is already known for some cancers such as *Helicobacter pylori* and gastric cancer [5]. Accumulating studies indicate that activation of Toll-like receptors (TLRs) promotes the development of cancer by activating nuclear factor-κB (NF-κΒ) pathway and the transducer signal transducer and activator of transcription 3 (STAT3) in colon, gastric and liver cancers [55, 56]. A recent study about lung microbiome and lung cancer demonstrated that the local microbiome of lung provoked inflammation associated with lung tumor and promoted the progress of lung cancer [57]. This study established mouse model of lung cancer and demonstrated that commensal bacteria stimulated Myd88-dependent IL-1β and IL-23 production from myeloid cells, inducing proliferation and activation of Vγ6 + Vδ1+ γδT cells that produced IL-17 and other effector molecules which promote inflammation and tumor cell proliferation. In other study, IL-6 was important for the processing that IL-17 enhanced the invasion of non-small cell lung cancer. In the present study, IL-6 or CRP were correlated with specific LRT microbime at variable levels, indicating that microbiome dysbiosis might over stimulated immunity system of mice and drove the inflammation.

The complementary functions of microbiome harbored inside and on the surface of the host body are vital to maintain the host physiological homeostasis [58]. Recently, many studies report that in similar environment, the function of microbiome is also similar but the composition of microbiome might have great difference [16, 17]. The Human Microbiome Project initiated by the National Institutes of Health (NIH) also shows that the function of microbiome remains relatively stable, despite dramatic variations in the community structure [18]. Li D studied the function of gastrointestinal microbiome of the rat. They found that small-molecule transport activity and amino acid metabolism were enriched in the upper Gastrointestinal Tract (GIT) and the mucolysis-related metabolism in the lower GIT. Moreover, the microbiome functions are similar although in different hosts [59]. But the studies about the functional prediction of microbiome in LRT were limited. Based on the PICRUSt (Phylogenetic Investigation of Communities by Reconstruction of Unobserved States), our study revealed that some function of microbiome in LRT altered under smoking condition. Among them, the ribosome, DNA repair and recombination proteins in KEGG3 level and Replication, recombination and repair in COG2 level were increased in smoking group which indicting that under chronic inflammation, the increased leukocytes produced reactive oxygen species, nitric oxide, metalloproteinases and interleukins that might promote the genomic instability and finally contribute to carcinogenesis. So the function relevant to DNA repair increased. We also found that transporters, ABC transporters which were responsible for the transportation and absorption of nutrients were significantly decreased. We speculated that smoking changed the pH, oxygen tension, temperature of LRT and this environment was not suitable for the living of some predominant microbiome. Noteworthy, in our study, the amino acid transport and metabolism de-regulated which was different from previous studies. Ionescu et al. reports that protein catabolism increased in CF patients, probably due to the destruction of cellular and connective tissue proteins, which is related to the degree of impaired lung function and the systemic inflammatory response [60]. Furthermore, the pathway involved in xenobiotic degeneration including dioxin was decreased when compared with normal mouse, which suggesting that exposed to the smoking for a long time would weaken the ability of degrading xenobiotic compounds. This was further demonstrated that the change of microbiome functions can influence the physiological homeostasis of lung.

To conclude, this study mainly investigated the variation of LRT microbial community composition and functions in smoking exposed mice and further explored the relationship between LRT microbiome and inflammation. The study demonstrated that smoking could change the microbial community composition and disturb the homeostasis of microbiome which also called microbiome dysbiosis. Furthermore, there existed associations between variation of lung microbiome and inflammation mediators, which indicated a potential correlation between LRT microbiome and immune system. Microbiome dysbiosis might break the balance of immune system to drive lung inflammation and the inflammation further promoted microbiome dysbiosis in LRT, causing a vicious cycle. And chronic inflammations promoted the process of variable pulmonary diseases. On the other hand, there might exist potential probiotics in LRT which were important for the maintain of physiological homeostasis of lung, such as *Lactobacillaceae.* Importantly, the function of *LRT m*icrobiome altered under smoking exposure, affecting the physiological homeostasis of host. Thus, it is essential to understand the effect of LRT microbiome on the development of lung disease and it provided a novel perspective for treating the pulmonary diseases. Of course, more studies are needed to further investigate the mechanisms that how LRT microbiome interact with host.

## Conclusions

Our study demonstrated that smoking could change the microbial community composition.There are associations between variation of lung microbiome and inflammation mediators. Microbiome dysbiosis might break the balance of immune system to drive lung inflammation and the inflammation further promoted microbiome dysbiosis in LRT, causing a vicious cycle, which indicating a potential correlation between microbiome and immune system.There might exist potential probiotics in Lower respiratory tract, such as Lactobacillaceae. The altered function of Lower respiratory tract microbiome under smoking exposure may affect the physiological homeostasis of host.This study provided a novel perspective for treating the pulmonary diseases.

## Supplementary information

**Additional file 1: Figure S1.** Number of OTUs that were considered as core microbiome. **Figure S2.** Taxonomy of the top30 genus. **Figure S3.** Distance within groups or among groups. **Figure S4.** Scatter diagram of IL-6 and *Lactobacillus* in smoking group. **Figure S5.** Scatter diagram of CRP and *Lactobacillus* in smoking group. **Figure S6.** Scatter diagram of IL-6 and *Deltaproteobacteria* in smoking group. **Figure S7.** Scatter diagram of IL-6 and *Oceanospirillales* in smoking group. **Figure S8.** Scatter diagram of IL-6 and *Lactobacillaceae* in smoking group. **Figure S9.** Scatter diagram of IL-6 and *Geobacteraceae* in smoking group. **Figure S10.** Scatter diagram of IL-6 and *Geobacter* in smoking group. **Figure S11.** Scatter diagram of IL-6 and *Nesterenkonia* in smoking group.

**Additional file 2: Table S1.** Table of ANOSIM analysis between smoking and non-smoking group. **Table S2.** Comparison of Phyla higher than 0.2% between smoking and non-smoking group. **Table S3.** Comparison of Class higher than 0.2% between smoking and non-smoking group. **Table S4.** Comparison of Order higher than 0.5% between smoking and non-smoking group. **Table S5.** Comparison of Family higher than 0.5% between smoking and non-smoking group. **Table S6.** Comparison of Genus higher than 0.5% between smoking and non-smoking group. **Table S7.** Comparison of Species higher than 0.5% between smoking and non-smoking group. **Table S8.** Spearman correlation coefficient between inflammatory mediators and Phyla higher than 0.2% in smoking and non-smoking group. **Table S9.** Spearman correlation coefficient between inflammatory mediators and Class higher than 0.2% in smoking and non-smoking group. **Table S10.** Spearman correlation coefficient between inflammatory mediators and Order higher than 0.5% in smoking and non-smoking group. **Table S11.** Spearman correlation coefficient between inflammatory mediators and Family higher than 0.5% in smoking and non-smoking group. **Table S12.** Spearman correlation coefficient between inflammatory mediators and Genus higher than 0.5% in smoking and non-smoking group. **Table S13.** Spearman correlation coefficient between inflammatory mediators and Species higher than 0.5% in smoking and non-smoking group.

## Data Availability

The datasets used and analysed during the current study are available from the corresponding author on reasonable request.

## Abbreviations

CF: Cystic fibrosis
COG: Clusters of Orthologous Groups of proteins
COPD: Chronic obstructive pulmonary disease
CRP: C-reactive protein
ELISA: Enzyme Linked Immunosorbent Assay
GIT: Gastrointestinal Tract
IL-6: Interleukin-6
IPF: Idiopathic pulmonary fibrosis
KEGG: Kyoto Encyclopedia of Genes and Genomes
LRT: Lower Respiratory Tract
OTUs: Operational Taxonomic Units
PICRUSt: Phylogenetic Investigation of Communities by Reconstruction of Unobserved States
TLRs: Toll-like receptors
