# Supplementary material for: Dysbiosis of lower respiratory tract microbiome are associated with inflammation and microbial function variety
Source: Respir Res. 2019 Dec 3;20:272. doi: 10.1186/s12931-019-1246-0 (PMC6892239; doi:10.1186/s12931-019-1246-0)
Supplement: Supplementary file 1 — Additional file 1: Figure S1. Number of OTUs that were considered as core microbiome. Figure S2. Taxonomy of the top30 genus. Figure S3. Distance within groups or among groups. Figure S4. Scatter diagram of IL-6 and Lactobacillus in smoking group. Figure S5. Scatter diagram of CRP and Lactobacillus in smoking group. Figure S6. Scatter diagram of IL-6 and Deltaproteobacteria in smoking group. Figure S7. Scatter diagram of IL-6 and Oceanospirillales in smoking group. Figure S8. Scatter diagram of IL-6 and Lactobacillaceae in smoking group. Figure S9. Scatter diagram of IL-6 and Geobacteraceae in smoking group. Figure S10. Scatter diagram of IL-6 and Geobacter in smoking group. Figure S11. Scatter diagram of IL-6 and Nesterenkonia in smoking group. [file 12931_2019_1246_MOESM1_ESM.docx]

##### Dysbiosis of lower respiratory tract microbiome are associated with inflammation and microbial function variety

Kang-jie Li^1#^, Zi-long Chen^2#^, Yao Huang^2^, Rui Zhang^1^, Xiao-qian Luan^1^, Ting-ting Lei^2^, Ling Chen^3*^

1 School of Public Health and Management, Chongqing Medical University, Chongqing 400016, China

2 First Clinical College, Chongqing Medical University, Chongqing 400016, China

3 The Center of Experimental Teaching Management, Chongqing Medical University, Chongqing 401331, China

#Kang-jie Li, first author, email:1539443009@qq.com, institutional address: School of Public Health and Management, Chongqing Medical University, Chongqing 400016, China

#Zi-long Chen, first author, email:857728299@qq.com, institutional address: First Clinical College, Chongqing Medical University, Chongqing 400016, China

Kang-jie Li and Zi-long Chen contributed equally to this work.

Yao Huang, email: 1154813475 @qq.com, institutional address: First Clinical College, Chongqing Medical University, Chongqing 400016, China

Rui Zhang, email: [renee1296@163.com,](mailto:renee1296@163.com,) institutional address: School of Public Health and Management, Chongqing Medical University, Chongqing 400016, China

Xiao-qian Luan, email: 1640006074 @qq.com, institutional address: School of Public Health and Management, Chongqing Medical University, Chongqing 400016, China

Ting-ting Lei, email: [862446198@qq.com,](mailto:862446198@qq.com,) institutional address: First Clinical College, Chongqing Medical University, Chongqing 400016, China

*Ling Chen, corresponding author, email: chenling@cqmu.edu.cn, institutional address: The Center of Experimental Teaching Management, Chongqing Medical University, Chongqing 401331, China


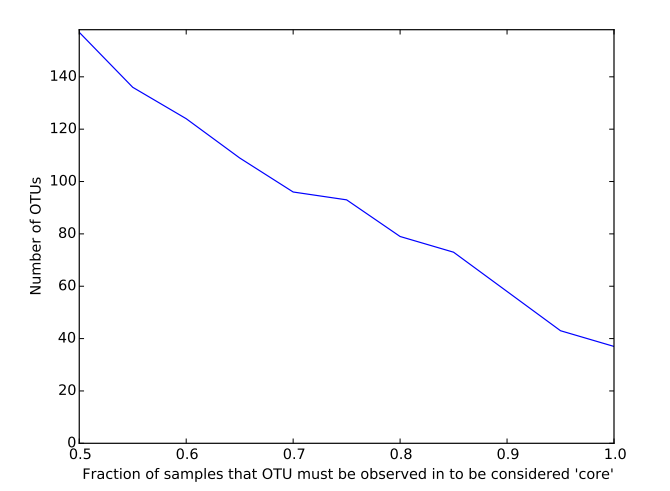


Figure S1. Number of OTUs that were considered as core microbiome.

**
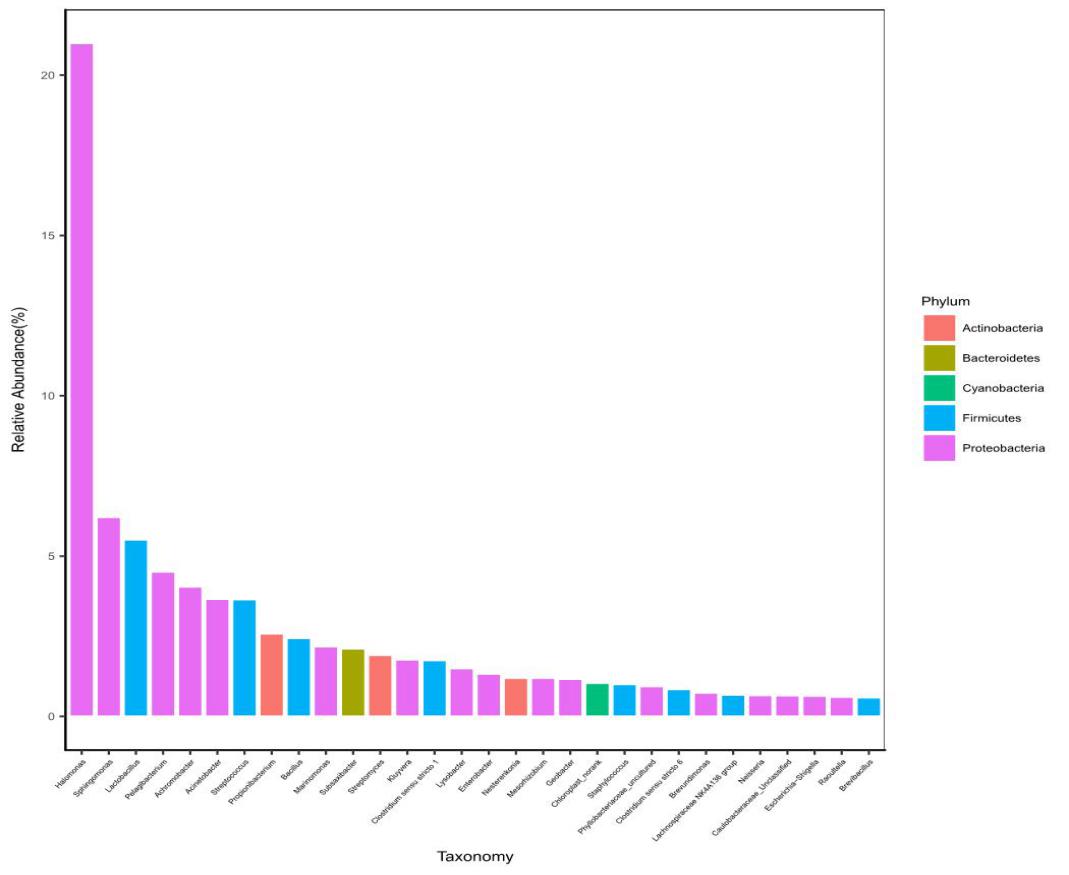
**

Figure S2. Taxonomy of the top30 genus. The top30 genus in both smoking group and non-smoking group can be classified into five phyla and each color indicates one phyla.

**A B C**

**
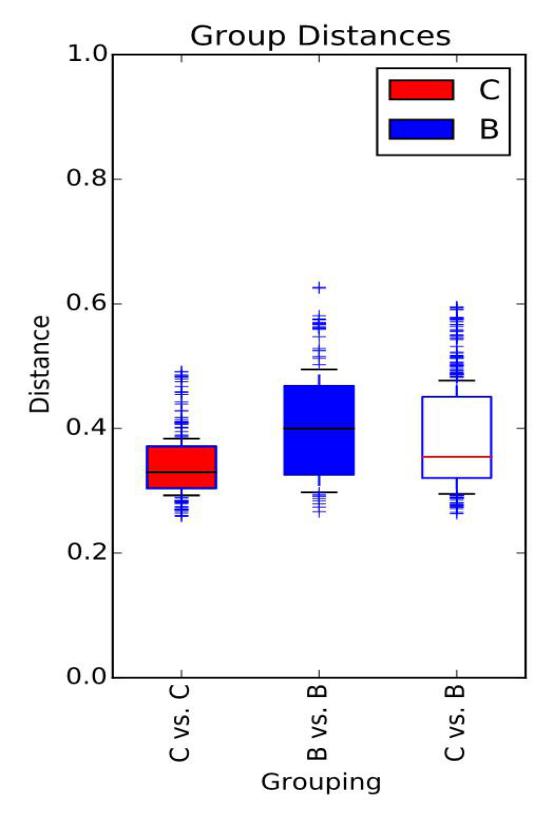

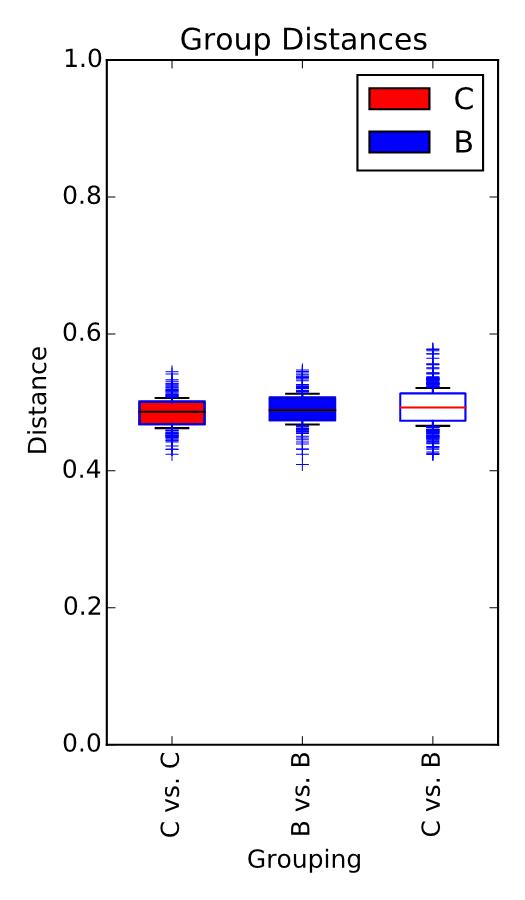

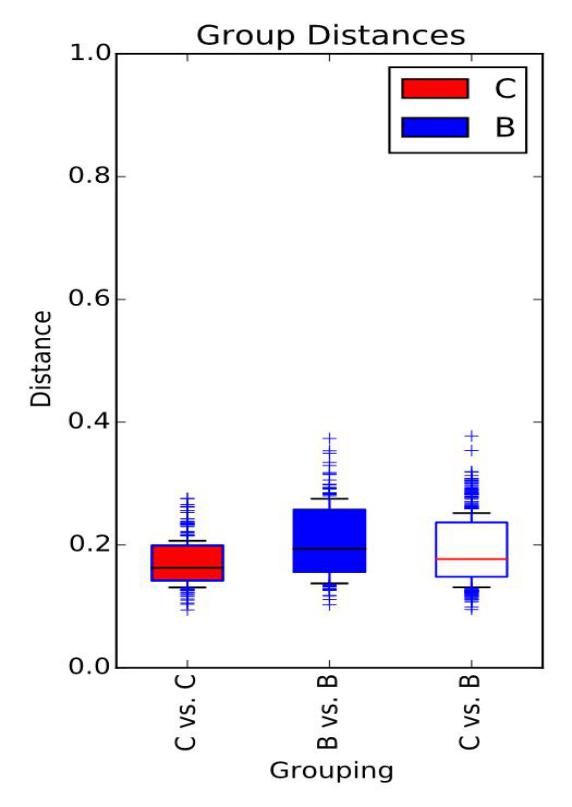
**

Figure S3. Distance within groups or among groups. **A** Distance within groups or among groups based on Bray-Curtis distance. **B** Distance within groups or among groups based on unweight uniFrac distance. **C** Distance within groups or among groups based on weighted uniFrac distance. Group B colored with blue indicates the smoking group and Group C colored with red indicates the non-smoking group.


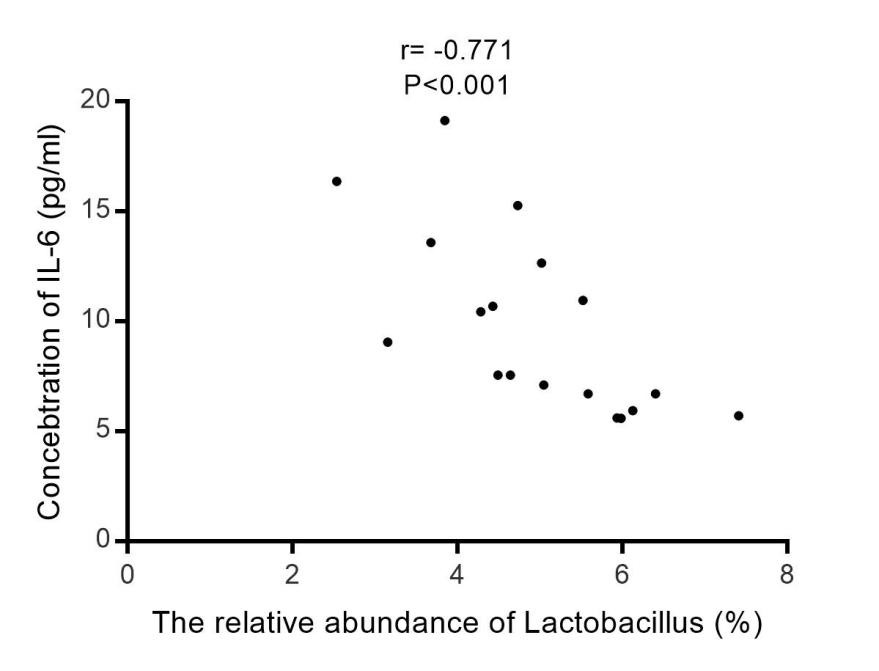


Figure S4. Scatter diagram of IL-6 and *Lactobacillus* in smoking group. r is the correlation coefficient. P-value < 0.001 indicates the significant association within IL-6 and *Lactobacillus*.


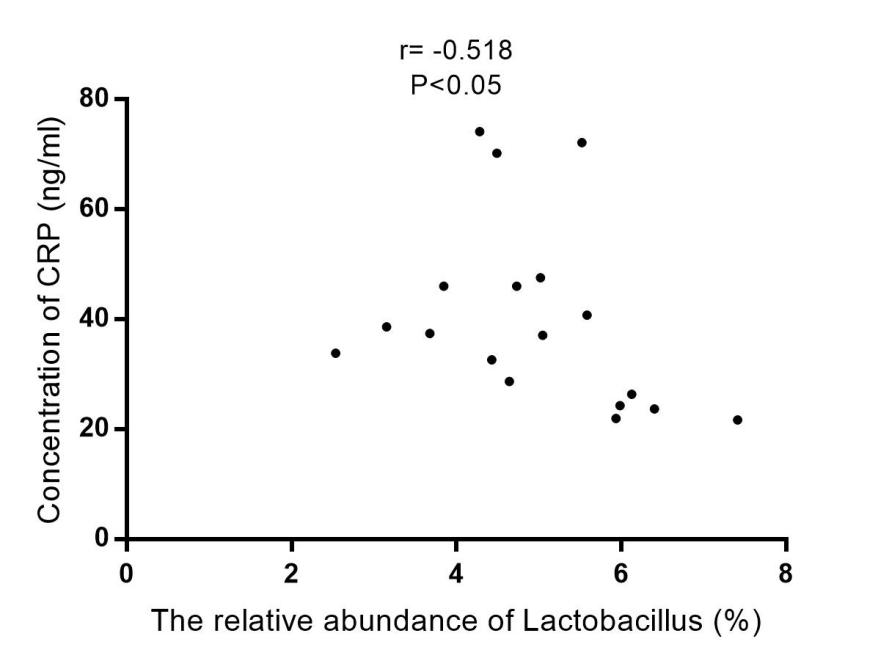


Figure S5. Scatter diagram of CRP and *Lactobacillus* in smoking group. r is the correlation coefficient. P-value < 0.05 indicates the significant association within CRP and *Lactobacillus*.


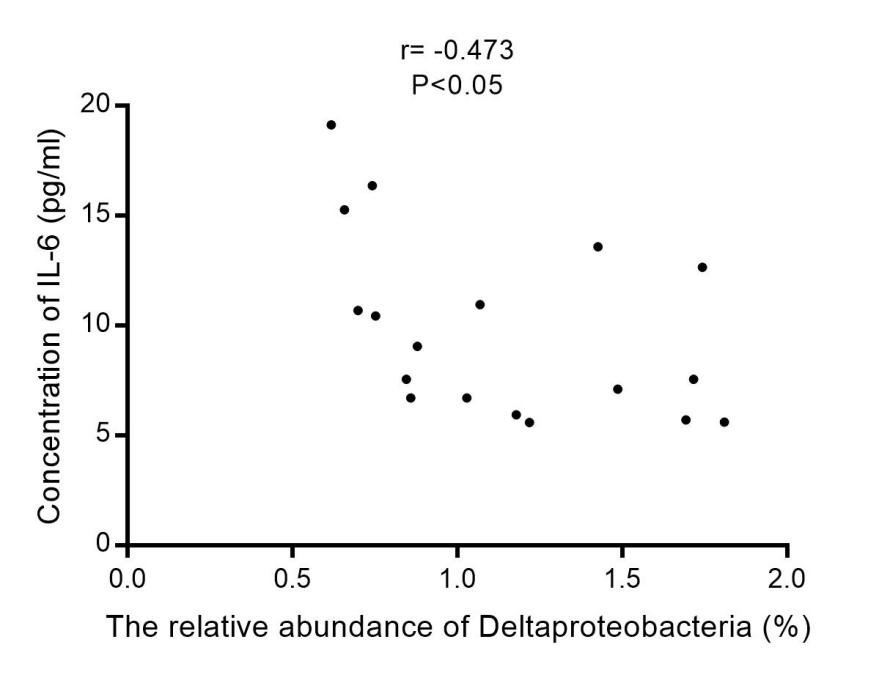


Figure S6. Scatter diagram of IL-6 and *Deltaproteobacteria* in smoking group. r is the correlation coefficient. P-value < 0.05 indicates the significant association within IL-6 and *Deltaproteobacteria*.


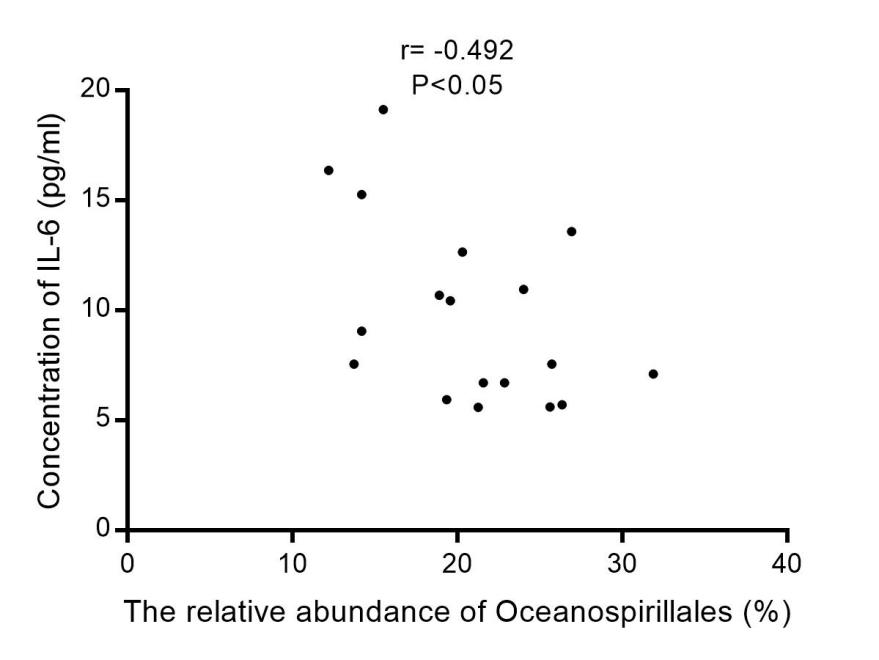


Figure S7. Scatter diagram of IL-6 and *Oceanospirillales* in smoking group. r is the correlation coefficient. P-value < 0.05 indicates the significant association within IL-6 and *Oceanospirillales*.


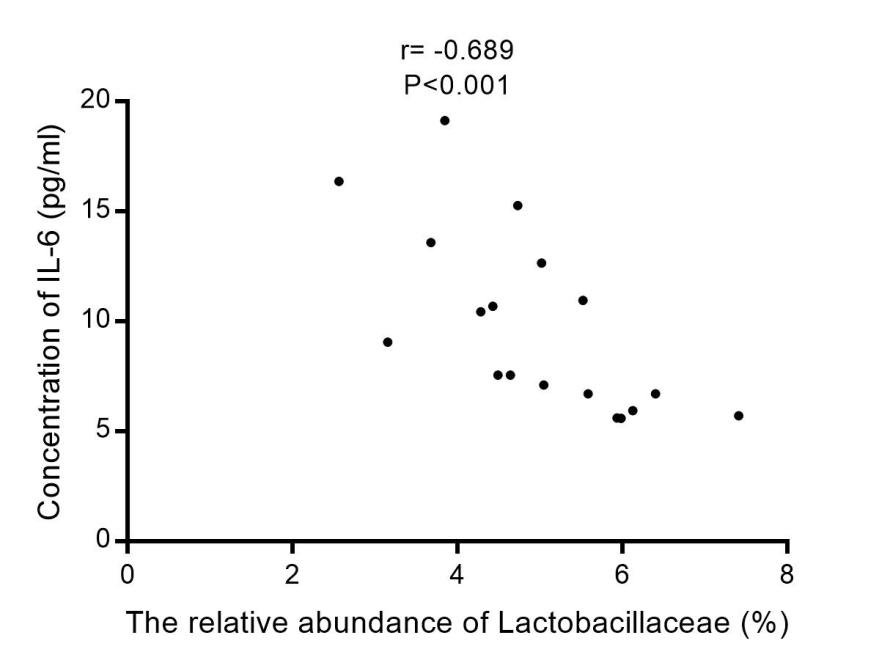


Figure S8. Scatter diagram of IL-6 and *Lactobacillaceae* in smoking group. r is the correlation coefficient. P-value < 0.001 indicates the significant association within IL-6 and *Lactobacillaceae*.


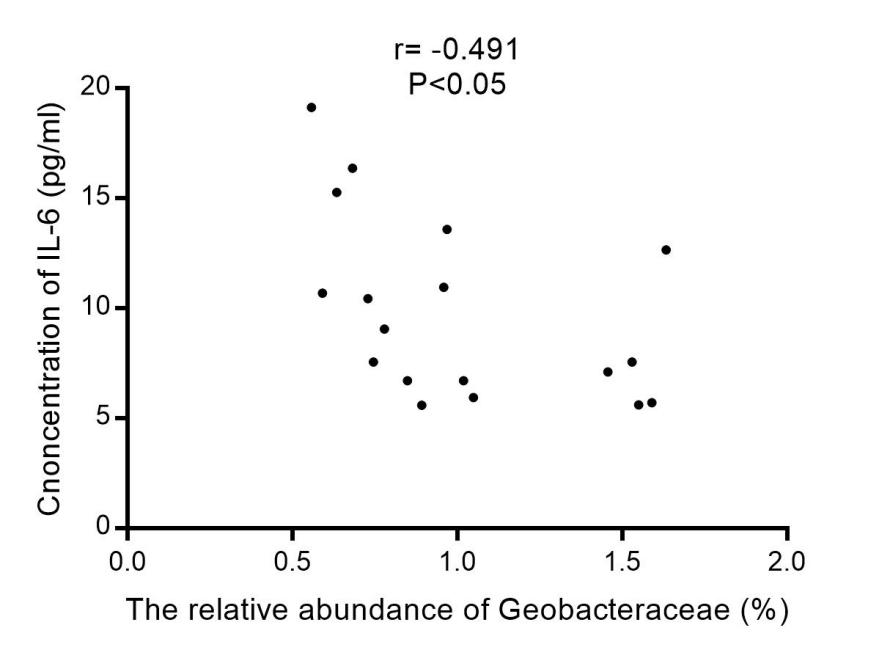


Figure S9. Scatter diagram of IL-6 and *Geobacteraceae* in smoking group. r is the correlation coefficient. P-value < 0.05 indicates the significant association within IL-6 and *Geobacteraceae*.


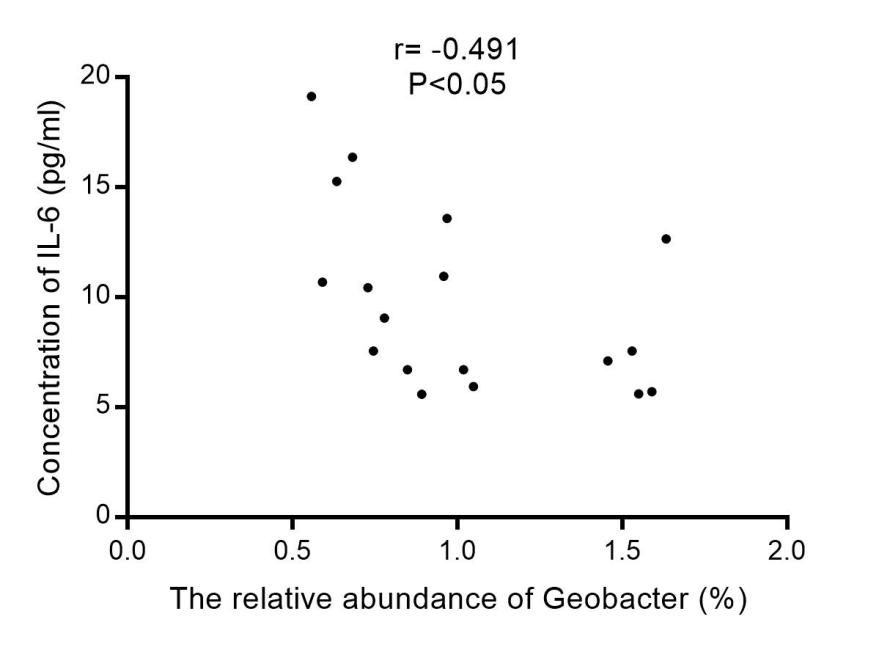


Figure S10. Scatter diagram of IL-6 and *Geobacter* in smoking group. r is the correlation coefficient. P-value < 0.05 indicates the significant association within IL-6 and *Geobacter*.


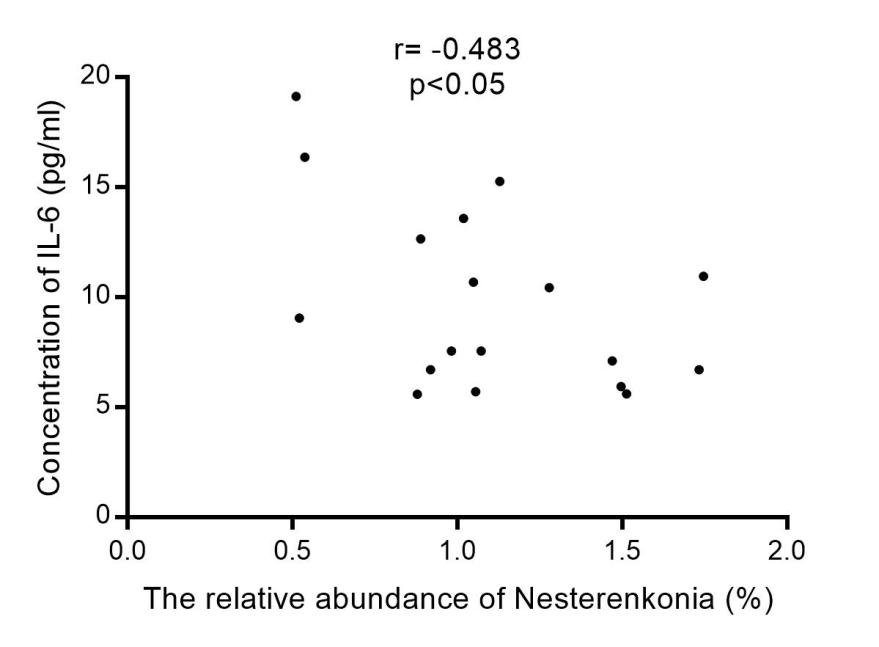


Figure S11. Scatter diagram of IL-6 and *Nesterenkonia* in smoking group. r is the correlation coefficient. P-value < 0.05 indicates the significant association within IL-6 and *Nesterenkonia*
