# Supplementary material for: Dysbiosis of lower respiratory tract microbiome are associated with inflammation and microbial function variety
Source: Respir Res. 2019 Dec 3;20:272. doi: 10.1186/s12931-019-1246-0 (PMC6892239; doi:10.1186/s12931-019-1246-0)
Supplement: Supplementary file 2 — Additional file 2: Table S1. Table of ANOSIM analysis between smoking and non-smoking group. Table S2. Comparison of Phyla higher than 0.2% between smoking and non-smoking group. Table S3. Comparison of Class higher than 0.2% between smoking and non-smoking group. Table S4. Comparison of Order higher than 0.5% between smoking and non-smoking group. Table S5. Comparison of Family higher than 0.5% between smoking and non-smoking group. Table S6. Comparison of Genus higher than 0.5% between smoking and non-smoking group. Table S7. Comparison of Species higher than 0.5% between smoking and non-smoking group. Table S8. Spearman correlation coefficient between inflammatory mediators and Phyla higher than 0.2% in smoking and non-smoking group. Table S9. Spearman correlation coefficient between inflammatory mediators and Class higher than 0.2% in smoking and non-smoking group. Table S10. Spearman correlation coefficient between inflammatory mediators and Order higher than 0.5% in smoking and non-smoking group. Table S11. Spearman correlation coefficient between inflammatory mediators and Family higher than 0.5% in smoking and non-smoking group. Table S12. Spearman correlation coefficient between inflammatory mediators and Genus higher than 0.5% in smoking and non-smoking group. Table S13. Spearman correlation coefficient between inflammatory mediators and Species higher than 0.5% in smoking and non-smoking group. [file 12931_2019_1246_MOESM2_ESM.docx]

##### Dysbiosis of lower respiratory tract microbiome are associated with inflammation and microbial function variety

Kang-jie Li^1#^, Zi-long Chen^2#^, Yao Huang^2^, Rui Zhang^1^, Xiao-qian Luan^1^, Ting-ting Lei^2^, Ling Chen^3*^

1 School of Public Health and Management, Chongqing Medical University, Chongqing 400016, China

2 First Clinical College, Chongqing Medical University, Chongqing 400016, China

3 The Center of Experimental Teaching Management, Chongqing Medical University, Chongqing 401331, China

#Kang-jie Li, first author, email:1539443009@qq.com, institutional address: School of Public Health and Management, Chongqing Medical University, Chongqing 400016, China

#Zi-long Chen, first author, email:857728299@qq.com, institutional address: First Clinical College, Chongqing Medical University, Chongqing 400016, China

Kang-jie Li and Zi-long Chen contributed equally to this work.

Yao Huang, email: 1154813475 @qq.com, institutional address: First Clinical College, Chongqing Medical University, Chongqing 400016, China

Rui Zhang, email: [renee1296@163.com,](mailto:renee1296@163.com,) institutional address: School of Public Health and Management, Chongqing Medical University, Chongqing 400016, China

Xiao-qian Luan, email: 1640006074 @qq.com, institutional address: School of Public Health and Management, Chongqing Medical University, Chongqing 400016, China

Ting-ting Lei, email: [862446198@qq.com,](mailto:862446198@qq.com,) institutional address: First Clinical College, Chongqing Medical University, Chongqing 400016, China

*Ling Chen, corresponding author, email: chenling@cqmu.edu.cn, institutional address: The Center of Experimental Teaching Management, Chongqing Medical University, Chongqing 401331, China

Table S1. Table of ANOSIM analysis between smoking and non-smoking group

on unweighted UniFrac distance on weighted UniFrac distance on Bray-Curtis distance

method name ANOSIM ANOSIM ANOSIM

test statistic name R R R

sample size 36 36 36

number of groups 2 2 2

test statistic 0.131 0.019 0.084

p-value 0.002* 0.202 0.005*

number of permutations 999 999 99**9**

*P-value < 0.05 is considered to be significant.

Table S2. Comparison of Phyla higher than 0.2% between smoking and non-smoking group.

| Phyla | Smoking group | | Non-smoking group | | P-value |
| --- | --- | --- | --- | --- | --- |
|  | Mean(%) | SD | Mean(%) | SD |  |
| Proteobacteria | 61.64 | 8.21 | 62.96 | 4.32 | 0.728 |
| Firmicutes | 24.00 | 8.23 | 23.51 | 3.39 | 0.393 |
| Actinobacteria | 7.07 | 1.80 | 6.54 | 2.84 | 0.174 |
| Bacteroidetes | 4.77 | 1.97 | 4.12 | 1.25 | 0.402 |
| Cyanobacteria | 0.92 | 0.73 | 1.68 | 3.08 | 0.591 |
| Acidobacteria | 0.47 | 0.43 | 0.26 | 0.19 | 0.205 |
| Planctomycetes | 0.25 | 0.20 | 0.16 | 0.13 | 0.274 |

The relative abundance of phyla higher than 0.2% in any group were shown.

Statistical analyses were performed using Wilcoxon rank-sum test.

Table S3.Comparison of Class higher than 0.2% between smoking and non-smoking group.

| Class | Smoking group | | Non-smoking group | | P-value |
| --- | --- | --- | --- | --- | --- |
|  | Mean(%) | SD | Mean(%) | SD |  |
| Gammaproteobacteria | 34.95 | 7.89 | 36.16 | 5.14 | 0.506 |
| Alphaproteobacteria | 17.59 | 3.25 | 17.70 | 2.70 | 0.899 |
| Bacilli | 15.85 | 6.80 | 15.34 | 3.17 | 0.537 |
| Betaproteobacteria | 7.81 | 2.06 | 7.58 | 1.32 | 0.849 |
| Clostridia | 7.31 | 1.88 | 7.53 | 1.51 | 0.448 |
| Actinobacteria | 6.81 | 1.80 | 6.35 | 2.82 | 0.211 |
| Flavobacteriia | 2.83 | 1.86 | 2.44 | 0.98 | 0.800 |
| Bacteroidia | 1.14 | 0.57 | 0.89 | 0.47 | 0.195 |
| Deltaproteobacteria | 1.13 | 0.41 | 1.43 | 0.35 | **0.029** |
| Sphingobacteriia | 0.67 | 0.41 | 0.65 | 0.44 | 0.764 |
| Chloroplast | 0.55 | 0.43 | 1.53 | 3.06 | **0.033** |
| Limnochordia | 0.47 | 1.78 | 0.11 | 0.11 | 0.071 |
| Cyanobacteria | 0.35 | 0.65 | 0.15 | 0.19 | 0.845 |
| Negativicutes | 0.32 | 0.37 | 0.44 | 0.30 | 0.141 |
| Planctomycetacia | 0.20 | 0.18 | 0.11 | 0.11 | 0.106 |

The relative abundance of class higher than 0.2% in any group were shown.

Statistical analyses were performed using Wilcoxon rank-sum test.

Table S4.Comparison of Order higher than 0.5% between smoking and non-smoking group.

| Order | Smoking group | | | Non-smoking group | | P-value | |
| --- | --- | --- | --- | --- | --- | --- | --- |
|  | Mean(%) | SD | Mean(%) | | SD |  |  |

| Oceanospirillales | 20.78 | 5.43 | 25.64 | 5.32 | **0.015** |
| --- | --- | --- | --- | --- | --- |
| Lactobacillales | 10.96 | 6.18 | 10.29 | 2.16 | 0.376 |
| Rhizobiales | 8.18 | 1.83 | 8.97 | 1.45 | 0.164 |
| Clostridiales | 7.23 | 1.86 | 7.50 | 1.50 | 0.467 |
| Sphingomonadales | 7.11 | 1.61 | 6.46 | 1.82 | 0.146 |
| Pseudomonadales | 6.26 | 7.80 | 2.60 | 0.92 | 0.121 |
| Burkholderiales | 6.10 | 1.69 | 5.67 | 1.09 | 0.393 |
| Bacillales | 4.81 | 3.52 | 5.06 | 2.52 | 0.411 |
| Enterobacteriales | 3.92 | 0.95 | 4.92 | 1.19 | **0.021** |
| Propionibacteriales | 3.05 | 1.53 | 2.21 | 1.34 | 0.094 |
| Xanthomonadales | 2.99 | 2.65 | 1.81 | 2.00 | 0.137 |
| Flavobacteriales | 2.83 | 1.86 | 2.44 | 0.98 | 0.800 |
| Streptomycetales | 1.65 | 1.22 | 2.17 | 1.85 | 0.335 |
| Micrococcales | 1.62 | 0.41 | 1.71 | 0.30 | 0.467 |
| Caulobacterales | 1.50 | 1.32 | 1.38 | 0.57 | 0.613 |
| Bacteroidales | 1.14 | 0.57 | 0.89 | 0.47 | 0.195 |
| Desulfuromonadales | 1.01 | 0.37 | 1.32 | 0.32 | **0.017** |
| Rhodocyclales | 0.72 | 0.38 | 0.89 | 0.35 | 0.133 |
| Sphingobacteriales | 0.67 | 0.41 | 0.65 | 0.44 | 0.764 |
| Neisseriales | 0.64 | 0.63 | 0.73 | 0.59 | 0.289 |
| Chloroplast_norank | 0.55 | 0.43 | 1.53 | 3.06 | **0.033** |
| Pasteurellales | 0.53 | 0.60 | 0.56 | 0.83 | 0.194 |

The relative abundance of order higher than 0.5% in any group were shown.

Statistical analyses were performed using Wilcoxon rank-sum test.

Table S5.Comparison of Family higher than 0.5% between smoking and non-smoking group.

| Family | Smoking group | | Non-smoking group | | P-value |
| --- | --- | --- | --- | --- | --- |
|  | Mean(%) | SD | Mean(%) | SD |  |
| Halomonadaceae | 20.01 | 5.44 | 20.02 | 3.99 | 0.282 |
| Sphingomonadaceae | 6.80 | 1.68 | 6.23 | 1.83 | 0.217 |
| Moraxellaceae | 5.83 | 7.45 | 2.37 | 0.91 | 0.088 |
| Lactobacillaceae | 4.94 | 1.22 | 6.10 | 1.51 | **0.018** |
| Hyphomicrobiaceae | 4.49 | 1.44 | 4.95 | 1.15 | 0.402 |
| Alcaligenaceae | 4.31 | 1.65 | 3.87 | 1.01 | 0.121 |
| Enterobacteriaceae | 3.92 | 0.95 | 4.92 | 1.19 | **0.021** |
| Streptococcaceae | 3.82 | 1.22 | 3.85 | 1.04 | 0.950 |
| Propionibacteriaceae | 3.02 | 1.54 | 2.19 | 1.34 | 0.107 |
| Xanthomonadaceae | 2.90 | 2.65 | 1.73 | 2.00 | 0.154 |
| Flavobacteriaceae | 2.70 | 1.83 | 2.33 | 0.97 | 0.728 |
| Bacillaceae | 2.65 | 2.63 | 2.39 | 0.93 | 0.429 |
| Clostridiaceae 1 | 2.53 | 0.63 | 2.82 | 0.94 | 0.537 |
| Phyllobacteriaceae | 2.07 | 0.68 | 2.67 | 0.39 | **0.002** |
| Lachnospiraceae | 1.75 | 0.64 | 1.89 | 0.96 | 0.704 |
| Streptomycetaceae | 1.65 | 1.22 | 2.17 | 1.85 | 0.335 |
| Caulobacteraceae | 1.50 | 1.32 | 1.38 | 0.57 | 0.613 |
| Micrococcaceae | 1.28 | 0.37 | 1.46 | 0.27 | 0.164 |
| Ruminococcaceae | 1.25 | 0.78 | 1.28 | 0.47 | 0.516 |
| Staphylococcaceae | 1.16 | 0.70 | 0.95 | 0.76 | 0.217 |
| Carnobacteriaceae | 1.05 | 3.53 | 0.16 | 0.17 | 0.183 |
| Geobacteraceae | 1.01 | 0.37 | 1.32 | 0.32 | **0.017** |
| Comamonadaceae | 0.92 | 0.46 | 0.95 | 0.37 | 0.788 |
| Peptostreptococcaceae | 0.81 | 0.56 | 1.06 | 0.50 | 0.164 |
| Enterococcaceae | 0.79 | 2.55 | 0.10 | 0.10 | 0.182 |
| Oceanospirillaceae | 0.76 | 0.69 | 3.60 | 7.12 | 0.097 |
| Bradyrhizobiaceae | 0.74 | 0.38 | 0.80 | 0.33 | 0.537 |
| Rhodocyclaceae | 0.72 | 0.38 | 0.89 | 0.35 | 0.133 |
| Neisseriaceae | 0.64 | 0.63 | 0.73 | 0.59 | 0.289 |
| Family XI | 0.59 | 1.00 | 0.27 | 0.26 | 0.467 |
| Chloroplast_norank | 0.55 | 0.43 | 1.53 | 3.06 | **0.033** |
| Pasteurellaceae | 0.53 | 0.60 | 0.56 | 0.83 | 0 .194 |
| Oxalobacteraceae | 0.52 | 0.35 | 0.25 | 0.23 | **0.018** |
| Burkholderiaceae | 0.36 | 0.28 | 0.59 | 0.40 | **0.033** |
| Paenibacillaceae | 0.30 | 0.30 | 1.12 | 0.21 | 0.090 |

The relative abundance of family higher than 0.5% in any group were shown.

Statistical analyses were performed using Wilcoxon rank-sum test.

Table S6.Comparison of Genus higher than 0.5% between smoking and non-smoking group.

| Genus | Smoking group | | Non-smoking group | | P-value |
| --- | --- | --- | --- | --- | --- |
|  | Mean(%) | SD | Mean(%) | SD |  |
| Halomonas | 18.86 | 7.15 | 21.98 | 4.00 | 0.184 |
| Sphingomonas | 6.11 | 2.21 | 5.99 | 1.69 | 0.448 |
| Acinetobacter | 4.83 | 7.60 | 2.69 | 0.86 | 0.776 |
| Lactobacillus | 4.60 | 1.64 | 6.70 | 1.51 | **0.007** |
| Achromobacter | 4. 05 | 1.88 | 3.84 | 1.03 | 0.223 |
| Pelagibacterium | 4.03 | 1.77 | 4.76 | 1.20 | 0.242 |
| Streptococcus | 3.43 | 1.46 | 3.70 | 1.05 | 0.862 |
| Propionibacterium | 2.82 | 1.68 | 2.18 | 1.34 | 0.229 |
| Bacillus | 2.49 | 2.67 | 2.32 | 0.95 | 0.327 |
| Subsaxibacter | 2.25 | 1.89 | 1.91 | 0.95 | 0.776 |
| Lysobacter | 1.75 | 2.00 | 1.11 | 1.47 | 0.392 |
| Streptomyces | 1.61 | 1.27 | 2.17 | 1.85 | 0.261 |
| Clostridium sensu stricto 1 | 1.60 | 0.73 | 1.81 | 0.75 | 0.635 |
| Kluyvera | 1.49 | 0.67 | 1.94 | 0.57 | **0.022** |
| Nesterenkonia | 1.05 | 0.46 | 1.30 | 0.27 | **0.046** |
| Staphylococcus | 1.05 | 0.76 | 0.88 | 0.71 | 0.384 |
| Mesorhizobium | 1.03 | 0.48 | 1.32 | 0.29 | **0.031** |
| Trichococcus | 0.99 | 3.50 | 0.09 | 1.71 | **0.030** |
| Geobacter | 0.96 | 0.44 | 1.32 | 0.32 | **0.015** |
| Brevundimonas | 0.86 | 1.22 | 0.56 | 0.45 | 0.527 |
| Enterobacter | 0.79 | 0.56 | 1.84 | 0.48 | **0.000** |
| Enterococcu | 0.79 | 2.55 | 0.10 | 0.10 | 0.215 |
| Escherichia-Shigella | 0.79 | 0.68 | 0.41 | 0.49 | **0.011** |
| Marinomonas | 0.74 | 0.70 | 3.60 | 7.12 | 0.079 |
| Phyllobacteriaceae_uncultured | 0.74 | 0.42 | 1.10 | 0.37 | **0.005** |
| Lachnospiraceae NK4A136 | 0.67 | 0.54 | 0.64 | 0.41 | 0.887 |
| Clostridium sensu stricto 6 | 0.64 | 0.39 | 0.98 | 0.53 | **0.018** |
| Neisseria | 0.60 | 0.63 | 0.69 | 0.60 | 0.248 |
| Caulobacteraceae_Unclassified | 0.51 | 0.28 | 0.77 | 0.25 | **0.012** |
| Chloroplast_norank | 0.51 | 0.45 | 1.53 | 3.06 | **0.017** |
| Raoultella | 0.48 | 0.28 | 0.70 | 0.22 | **0.004** |
| Zoogloea | 0.45 | 0.29 | 0.55 | 0.23 | 0.137 |
| Terrisporobacter | 0.40 | 0.40 | 0.57 | 0.32 | 0.058 |
| Brevibacillus | 0.15 | 0.12 | 1.01 | 2.21 | **0.007** |

The relative abundance of genus higher than 0.5% in any group were shown.

Statistical analyses were performed using Wilcoxon rank-sum test.

Table S7.Comparison of Species higher than 0.5% between smoking and non-smoking group.

| Species | Smoking group | | Non-smoking group | | P-value |
| --- | --- | --- | --- | --- | --- |
|  | Mean(%) | SD | Mean(%) | SD |  |
| Halomonas_Unclassified | 19.85 | 5.39 | 21.81 | 3.98 | 0.282 |
| Sphingomonas_uncultured bacterium | 5.49 | 1.47 | 5.08 | 1.43 | 0.486 |
| Acinetobacter_Unclassified | 5.22 | 7.54 | 2.03 | 0.86 | 0.376 |
| Pelagibacterium_uncultured bacterium | 4.26 | 1.47 | 4.76 | 1.20 | 0.359 |
| Achromobacter_Unclassified | 4.24 | 1.60 | 3.84 | 1.03 | 0.117 |
| Lactobacillus_uncultured bacterium | 3.34 | 0.97 | 4.00 | 1.35 | 0.179 |
| Propionibacterium_uncultured bacterium | 2.98 | 1.53 | 2.18 | 1.34 | 0.107 |
| Subsaxibacter_uncultured bacterium | 2.32 | 1.83 | 1.91 | 0.95 | 0.681 |
| Streptococcus gallolyticus subsp. Macedonicus | 2.27 | 0.76 | 2.81 | 0.65 | **0.023** |
| Lysobacter_uncultured bacterium | 1.87 | 1.96 | 1.11 | 1.47 | 0.229 |
| Streptomyces_Unclassified | 1.65 | 1.22 | 2.17 | 1.85 | 0.335 |
| Clostridium sensu stricto 1_uncultured bacterium | 1.62 | 0.58 | 1.81 | 0.74 | 0.681 |
| Kluyvera ascorbata | 1.59 | 0.56 | 1.94 | 0.57 | **0.041** |
| Bacillus_Unclassified | 1.50 | 0.76 | 1.83 | 0.77 | 0.155 |
| Lactobacillus_Unclassified | 1.33 | 0.44 | 1.47 | 0.34 | 0.282 |
| Streptococcus_uncultured bacterium | 1.22 | 0.66 | 0.82 | 0.71 | **0.037** |
| Staphylococcus_uncultured bacterium | 1.12 | 0.71 | 0.88 | 0.71 | 0.189 |
| Mesorhizobium_Unclassified | 1.06 | 0.43 | 1.28 | 0.27 | **0.043** |
| Nesterenkonia sp. NP1 | 1.00 | 0.37 | 1.21 | 0.28 | 0.054 |
| Trichococcus_uncultured bacterium | 1.00 | 3.50 | 0.09 | 1.71 | **0.027** |
| Sphingomonas_Unclassified | 0.94 | 0.65 | 0.90 | 0.36 | 0.752 |
| Geobacter_Unclassified | 0.90 | 0.38 | 1.06 | 0.24 | 0.121 |
| Escherichia-Shigella_Unclassified | 0.87 | 0.67 | 0.41 | 0.49 | **0.003** |
| Enterobacter_Unclassified | 0.82 | 0.53 | 1.84 | 0.48 | **0.000** |
| Enterococcus faecium NRRL B-2354 | 0.79 | 2.55 | 0.10 | 0.10 | 0.182 |
| Phyllobacteriaceae_uncultured bacterium | 0.77 | 0.38 | 1.10 | 0.37 | **0.005** |
| Marinomonas_Unclassified | 0.76 | 0.69 | 3.60 | 7.12 | 0.097 |
| Clostridium bornimense | 0.71 | 0.40 | 0.98 | 0.53 | 0.060 |
| Lachnospiraceae NK4A136 group_uncultured bacterium | 0.62 | 0.50 | 0.59 | 0.37 | 0.975 |
| Neisseria_uncultured bacterium | 0.61 | 0.62 | 0.69 | 0.60 | 0.255 |
| Bacillus_uncultured bacterium | 0.61 | 2.13 | 0.10 | 0.13 | 0.543 |
| Chloroplast_Unclassified | 0.54 | 0.44 | 1.50 | 3.06 | **0.041** |
| Brevundimonas nasdae | 0.54 | 1.21 | 0.37 | 0.30 | 0.548 |
| Caulobacteraceae_Unclassified | 0.53 | 0.25 | 0.77 | 0.25 | **0.013** |
| Raoultella_Unclassified | 0.51 | 0.25 | 0.70 | 0.22 | **0.007** |
| Zoogloea_uncultured bacterium | 0.48 | 0.27 | 0.55 | 0.23 | 0.242 |
| Terrisporobacter_uncultured bacterium | 0.43 | 0.38 | 0.57 | 0.32 | 0.114 |

The relative abundance of species higher than 0.5% in any group were shown.

Statistical analyses were performed using Wilcoxon rank-sum test.

Table S8. Spearman correlation coefficient between inflammatory mediators and Phyla higher than 0.2% in smoking and non-smoking group.

| Phyla | Smoking group | | Non-smoking group | |
| --- | --- | --- | --- | --- |
|  | IL-6 | CRP | IL-6 | CRP |
| Firmicutes | 0.183 | 0.045 | -0.02 | 0.082 |
| Protaobacteria | -0.19 | -0.152 | -0.22 | -0.057 |
| Bacteroidetes | -0.315 | -0.01 | -0.117 | -0.062 |
| Cyanobacteria | -0.306 | -0.024 | 0.274 | -0.017 |
| Acidobacteria | -0.024 | **0.521*** | 0.159 | 0.014 |
| Planctomycetes | -0.165 | **0.651*** | -0.155 | 0.169 |
| Bacteroidetes | -0.315 | -0.01 | -0.117 | -0.062 |

The bold numbers represent a statistical correlation.

*P-value < 0.05 is considered to be significant.

Table S9.Spearman correlation coefficient between inflammatory mediators and Class higher than 0.2% in smoking and non-smoking group.

| Class | Smoking group | | Non-smoking group | |
| --- | --- | --- | --- | --- |
|  | IL-6 | CRP | IL-6 | CRP |
| Gammaproteobacteria | 0.099 | -0.092 | -0.173 | 0.013 |
| Alphaproteobacteria | -0.238 | 0.137 | -0.071 | -0.149 |
| Bacilli | 0.238 | -0.146 | -0.045 | 0.03 |
| Betaproteobacteria | -0.386 | 0.007 | 0.239 | 0.069 |
| Clostridia | -0.061 | 0.401 | 0.138 | 0.194 |
| Actinobacteria | 0.106 | 0.137 | -0.035 | -0.075 |
| Flavobacteriia | -0.211 | 0.037 | 0.002 | -0.176 |
| Bacteroidia | -0.049 | -0.045 | -0.274 | 0.396 |
| Deltaproteobacteria | **-0.473*** | -0.4 | -0.207 | -0.166 |
| Sphingobacteriia | -0.427 | -0.135 | -0.011 | -0.131 |
| Chloroplast | -0.042 | 0.201 | 0.256 | -0.027 |
| Limnochordia | 0.07 | -0.1 | -0.299 | -0.017 |
| Cyanobacteria | -0.32 | -0.212 | 0.319 | 0.156 |
| Negativicutes | 0.175 | 0.151 | **0.562*** | **0.535*** |
| Planctomycetacia | -0.171 | 0.584 | -0.297 | -0.107 |

The bold numbers represent a statistical correlation.

*P-value < 0.05 is considered to be significant.

Table S10. Spearman correlation coefficient between inflammatory mediators and Order higher than 0.5% in smoking and non-smoking group.

| Order | Smoking group | | Non-smoking group | |
| --- | --- | --- | --- | --- |
|  | IL-6 | CRP | IL-6 | CRP |
| Oceanospirillales | **-0.492*** | -0.283 | -0.06 | 0.188 |
| Lactobacillales | 0.264 | -0.104 | -0.159 | -0.213 |
| Rhizobiales | -0.267 | 0.027 | -0.064 | -0.082 |
| Clostridiales | -0.058 | 0.411 | 0.132 | 0.182 |
| Sphingomonadales | -0.021 | 0.03 | -0.055 | -0.095 |
| Pseudomonadales | 0.357 | -0.065 | -0.189 | -0.344 |
| Burkholderiales | -0.234 | -0.039 | 0.359 | 0.205 |
| Bacillales | -0.007 | -0.09 | 0.079 | 0.221 |
| Enterobacteriales | -0.065 | -0.084 | -0.1 | -0.162 |
| Propionibacteriales | -0.049 | 0.253 | 0.096 | 0.145 |
| Xanthomonadales | 0.321 | 0.436 | -0.135 | -0.074 |
| Flavobacteriales | -0.211 | 0.037 | 0.002 | -0.176 |
| Streptomycetales | -0.059 | -0.051 | -0.046 | -0.187 |
| Micrococcales | -0.121 | -0.038 | -0.381 | -0.044 |
| Caulobacterales | -0.129 | 0.412 | -0.011 | -0.215 |
| Bacteroidales | -0.049 | -0.045 | -0.274 | 0.396 |
| Desulfuromonadales | **-0.491*** | -0.351 | -0.217 | -0.202 |
| Rhodocyclales | **-0.536*** | -0.246 | 0.275 | 0.32 |
| Sphingobacteriales | -0.427 | -0.135 | -0.011 | -0.131 |
| Neisseriales | -0.251 | 0.283 | -0.199 | -0.275 |
| Chloroplast_norank | -0.042 | 0.201 | 0.256 | -0.027 |
| Pasteurellales | -0.057 | 0.349 | -0.19 | -0.254 |

The bold numbers represent a statistical correlation.

*P-value < 0.05 is considered to be significant.

Table S11. Spearman correlation coefficient between inflammatory mediators and Family higher than 0.5% in smoking and non-smoking group.

| Family | Smoking group | | Non-smoking group | |
| --- | --- | --- | --- | --- |
|  | IL-6 | CRP | IL-6 | CRP |
| Halomonadaceae | **-0.497*** | -0.24 | 0.106 | 0.059 |
| Sphingomonadaceae | -0.09 | -0.056 | -0.032 | -0.113 |
| Moraxellaceae | 0.354 | -0.06 | -0.213 | -0.39 |
| Lactobacillaceae | **-0.689*** | -0.333 | -0.251 | -0.306 |
| Hyphomicrobiaceae | -0.435 | -0.156 | -0.154 | 0.069 |
| Alcaligenaceae | -0.325 | -0.01 | 0.397 | 0.163 |
| Enterobacteriaceae | -0.065 | -0.084 | -0.1 | -0.162 |
| Streptococcaceae | -0.301 | 0.159 | 0.071 | -0.059 |
| Propionibacteriaceae | -0.057 | 0.251 | 0.098 | 0.146 |
| Xanthomonadaceae | 0.321 | 0.439 | -0.118 | -0.078 |
| Flavobacteriaceae | -0.193 | 0.063 | 0.067 | -0.155 |
| Bacillaceae | -0.046 | -0.057 | 0.206 | 0.04 |
| Clostridiaceae 1 | 0.021 | 0.343 | -0.162 | -0.032 |
| Phyllobacteriaceae | -0.408 | -0.07 | 0.065 | -0.256 |
| Lachnospiraceae | -0.441 | -0.092 | 0.118 | -0.009 |
| Streptomycetaceae | -0.059 | -0.051 | -0.046 | -0.187 |
| Caulobacteraceae | -0.129 | 0.412 | -0.011 | -0.215 |
| Micrococcaceae | -0.431 | 0.064 | -0.384 | 0.076 |
| Ruminococcaceae | -0.083 | **0.509*** | 0.078 | 0.253 |
| Staphylococcaceae | 0.004 | -0.196 | -0.162 | 0.022 |
| Carnobacteriaceae | 0.415 | -0.085 | -0.135 | 0.018 |
| Geobacteraceae | **-0.491*** | -0.351 | -0.217 | -0.202 |
| Comamonadaceae | 0.083 | -0.149 | -0.186 | 0.057 |
| Peptostreptococcaceae | 0.155 | 0.31 | 0.167 | 0.346 |
| Enterococcaceae | 0.413 | -0.052 | 0.236 | 0.285 |
| Oceanospirillaceae | 0.053 | -0.327 | -0.103 | 0.107 |
| Bradyrhizobiaceae | -0.211 | 0.207 | 0.138 | -0.221 |
| Rhodocyclaceae | **-0.536*** | -0.246 | 0.275 | 0.32 |
| Neisseriaceae | -0.251 | 0.283 | -0.199 | -0.275 |
| Family XI | 0.11 | 0 | 0.191 | 0.044 |
| Chloroplast_norank | -0.042 | 0.201 | 0.256 | -0.027 |
| Pasteurellaceae | -0.057 | 0.349 | -0.19 | -0.254 |
| Oxalobacteraceae | 0.387 | -0.069 | 0.1 | 0.19 |
| Burkholderiaceae | -0.136 | 0.161 | 0.084 | -0.021 |
| Paenibacillaceae | -0.038 | -0.012 | 0.068 | 0.244 |

The bold numbers represent a statistical correlation.

*P-value < 0.05 is considered to be significant.

Table S12. Spearman correlation coefficient between inflammatory mediators and Genus higher than 0.5% in smoking and non-smoking group.

| Genus | Smoking group | | Non-smoking group | |
| --- | --- | --- | --- | --- |
|  | IL-6 | CRP | IL-6 | CRP |
| Halomonas | -0.423 | -0.347 | -0.15 | -0.127 |
| Sphingomonas | -0.031 | 0.019 | 0.036 | -0.108 |
| Acinetobacter | -0.148 | -0.145 | -0.112 | -0.393 |
| Lactobacillus | **-0.771*** | **-0.518*** | -0.088 | -0.214 |
| Pelagibacterium | -0.425 | -0.208 | -0.302 | -0.267 |
| Achromobacter | -0.111 | 0.186 | 0.467 | 0.399 |
| Streptococcus | -0.114 | 0.128 | 0.193 | -0.013 |
| Propionibacterium | -0.049 | 0.346 | -0.044 | 0.042 |
| Bacillus | -0.041 | 0.176 | 0.22 | 0.127 |
| Subsaxibacter | -0.188 | 0.081 | -0.002 | -0.174 |
| Lysobacter | 0.28 | 0.438 | -0.111 | -0.043 |
| Streptomyces | -0.059 | -0.05 | -0.046 | -0.196 |
| Clostridium sensu stricto 1 | -0.28 | **0.504*** | -0.302 | -0.101 |
| Kluyvera | -0.14 | -0.077 | -0.117 | -0.131 |
| Nesterenkonia | **-0.483*** | 0.055 | -0.428 | 0.029 |
| Staphylococcus | 0.007 | -0.198 | -0.169 | 0.056 |
| Mesorhizobium | -0.264 | 0.003 | 0.257 | 0.127 |
| Trichococcus | 0.407 | -0.085 | -0.225 | 0.088 |
| Geobacter | **-0.491*** | -0.351 | -0.217 | -0.202 |
| Brevundimonas | -0.13 | 0.385 | 0.075 | -0.095 |
| Enterobacter | -0.214 | 0.263 | -0.196 | -0.276 |
| Enterococcus | 0.413 | -0.052 | 0.236 | 0.285 |
| Escherichia-Shigella | 0.221 | -0.192 | -0.113 | -0.066 |
| Marinomonas | 0.134 | -0.31 | 0.253 | 0.158 |
| Phyllobacteriaceae_uncultured | -0.363 | -0.063 | -0.068 | -0.279 |
| Lachnospiraceae NK4A136 group | -0.187 | -0.101 | 0.044 | -0.065 |
| Clostridium sensu stricto 6 | -0.016 | -0.214 | 0.158 | 0.102 |
| Neisseria | -0.245 | 0.278 | -0.159 | -0.239 |
| Caulobacteraceae_Unclassified | -0.04 | 0.22 | -0.143 | -0.278 |
| Chloroplast_norank | -0.042 | 0.201 | 0.256 | -0.027 |
| Raoultella | -0.191 | -0.255 | 0.431 | 0.17 |
| Zoogloea | -0.446 | -0.282 | 0.291 | 0.459 |
| Terrisporobacter | 0.312 | 0.154 | 0.334 | 0.167 |
| Brevibacillus | -0.452 | -0.123 | 0.045 | 0.248 |

The bold numbers represent a statistical correlation.

*P-value < 0.05 is considered to be significant.

Table S13.Spearman correlation coefficient between inflammatory mediators and Species higher than 0.5% in smoking and non-smoking group.

| Species | Smoking group | | Non-smoking group | |
| --- | --- | --- | --- | --- |
|  | IL-6 | CRP | IL-6 | CRP |
| Halomonas_Unclassified | **-0.499*** | -0.24 | 0.101 | 0.056 |
| Sphingomonas_uncultured bacterium | -0.235 | -0.063 | -0.037 | -0.14 |
| Acinetobacter_Unclassified | 0.308 | -0.059 | -0.176 | -0.421 |
| Pelagibacterium_uncultured bacterium | -0.437 | -0.172 | -0.118 | 0.09 |
| Achromobacter_Unclassified | -0.342 | -0.01 | 0.365 | 0.16 |
| Lactobacillus_uncultured bacterium | **-0.672*** | -0.364 | -0.356 | -0.424 |
| Propionibacterium_uncultured bacterium | -0.061 | 0.258 | 0.096 | 0.142 |
| Subsaxibacter_uncultured bacterium | -0.188 | 0.079 | -0.002 | -0.175 |
| Streptococcus gallolyticus subsp. macedonicus | -0.291 | -0.151 | 0.373 | 0.018 |
| Lysobacter_uncultured bacterium | 0.287 | 0.442 | -0.111 | -0.044 |
| Streptomyces_Unclassified | -0.06 | -0.052 | -0.046 | -0.19 |
| Clostridium sensu stricto 1_uncultured bacterium | -0.318 | 0.32 | -0.314 | -0.101 |
| Kluyvera ascorbata | -0.14 | -0.08 | -0.117 | -0.128 |
| Bacillus_Unclassified | -0.328 | 0.067 | 0.336 | 0.036 |
| Lactobacillus_Unclassified | -0.417 | -0.082 | -0.124 | -0.088 |
| Streptococcus_uncultured bacterium | -0.107 | 0.413 | -0.277 | -0.155 |
| Staphylococcus_uncultured bacterium | 0.007 | -0.203 | -0.169 | 0.055 |
| Mesorhizobium_Unclassified | -0.245 | 0.002 | 0.37 | 0.187 |
| Nesterenkonia sp. NP1 | **-0.495*** | -0.023 | **-0.479*** | -0.018 |
| Trichococcus_uncultured bacterium | 0.407 | -0.085 | -0.225 | 0.088 |
| Sphingomonas_Unclassified | 0.416 | 0.012 | 0.025 | -0.013 |
| Geobacter_Unclassified | **-0.525*** | -0.43 | -0.027 | -0.211 |
| Escherichia-Shigella_Unclassified | 0.221 | -0.192 | -0.113 | -0.066 |
| Enterobacter_Unclassified | -0.214 | 0.263 | -0.196 | -0.276 |
| Enterococcus faecium NRRL B-2354 | 0.413 | -0.052 | 0.236 | 0.285 |
| Phyllobacteriaceae_uncultured bacterium | -0.363 | -0.063 | -0.076 | -0.276 |
| Marinomonas_Unclassified | 0.053 | -0.327 | -0.104 | 0.107 |
| Clostridium bornimense | -0.016 | -0.214 | 0.158 | 0.102 |
| Lachnospiraceae NK4A136 group_uncultured bacterium | -0.148 | -0.105 | 0.122 | 0.044 |
| Neisseria_uncultured bacterium | -0.244 | 0.277 | -0.156 | -0.236 |
| Bacillus_uncultured bacterium | 0.057 | -0.104 | 0.079 | -0.246 |
| Chloroplast_Unclassified | -0.028 | 0.197 | 0.244 | -0.035 |
| Brevundimonas nasdae | -0.204 | 0.4 | 0.212 | -0.077 |
| Caulobacteraceae_Unclassified | -0.037 | 0.258 | -0.143 | -0.278 |
| Raoultella_Unclassified | -0.191 | -0.255 | 0.431 | 0.17 |
| Zoogloea_uncultured bacterium | -0.446 | -0.282 | 0.291 | 0.459 |
| Terrisporobacter_uncultured bacterium | 0.312 | 0.154 | 0.334 | 0.167 |

The bold numbers represent a statistical correlation.

*P-value < 0.05 is considered to be significant.
